# Prevalence and associated factors of myopia among school students in Shenyang, China: a cross-sectional study

**DOI:** 10.3389/fpubh.2023.1239158

**Published:** 2023-08-30

**Authors:** Dan Zhang, Baijun Sun, Ming Wu, Huiying Liu, Lin Zhou, Lianying Guo

**Affiliations:** ^1^School of Public Health, Shenyang Medical College, Shenyang, China; ^2^Shenyang Center for Disease Control and Prevention, Shenyang, China; ^3^Liaoning Center for Disease Control and Prevention, Shenyang, China

**Keywords:** myopia, students, prevalence, associated factors, Shenyang

## Abstract

**Background:**

In recent years, the prevalence of myopia has increased significantly and caused great concern. Nevertheless, an estimate of myopia in the student population in Shenyang, Liaoning Province, China is still lacking. This study aims to determine the prevalence of myopia among students in Shenyang and investigate the associated factors affecting myopia development.

**Methods:**

Standard logarithmic visual acuity chart and automatic computerized optometry under non-ciliary muscle paralysis were used to test the students’ naked visual acuity of their right and left eyes. The included students were organized to fill in questionnaires on WeChat to collect the factors affecting myopia.

**Results:**

A total of 34,644 students with a median age of 11.9 years were examined, including 17,563 males and 17,081 females. The overall prevalence of myopia was 60%, with a prevalence of 45% for mild myopia, 13% for moderate myopia, and 1.9% for high myopia. The sex, high educational stage, family history of myopia, doing homework after school or reading and writing for more than 2 h were associated with a higher risk of myopia, while doing eye exercises twice a day or more, going outdoors during recess, reading and writing with eyes more than one foot from books, and sleeping more than 8 h a day were associated factors for preventing myopia. The associated factors influencing myopia vary among different subgroups.

**Conclusion:**

The prevalence of myopia in Shenyang is at a high level. In addition to sex, high educational stage and genetic factors, environmental factors including length of eye usage, eye exercises, outdoor activities, eye working distance, and sleep duration are associated with myopia prevalence. Therefore, it is recommended that the occurrence and development of myopia can be prevented by controlling the above environmental factors.

## 1. Introduction

Myopia is a visual condition characterized by the ability to see objects at close range clearly, but blurred or out of focus for distant objects. It is one of the most common eye diseases in the world and affects people of all ages and races (1, 2). Myopia can develop during childhood or adolescence and not only affects life but may also cause a variety of ocular diseases such as retinal detachment, macular degeneration and glaucoma, which can lead to blindness in severe cases (3, 4). In addition, some studies have shown that myopia may also be associated with the risk of developing other health problems such as cardiovascular disease and diabetes (5, 6).

The prevalence of myopia continues to rise worldwide with changes in people’s living and learning styles, and shows a trend toward younger and younger age of prevalence and increasing prevalence of high myopia, which means that myopia has become a major global public health problem (7, 8). Holden et al. (9) predicted that 4.758 billion people would have myopia by 2050, about half of the world’s population, and that East and Southeast Asia were expected to have the highest rates of myopia (10–12).

Understanding the prevalence of myopia and the factors that influence myopia is critical to developing effective prevention and treatment strategies. Although many studies have investigated the prevalence and associated factors for myopia, some factors remain controversial due to the complex interaction between genetic and environmental factors in the development of myopia, such as the inconsistent relationship between eye working distance and myopia (13, 14) and the inconsistent relationship between sleep and myopia (15–17). Therefore, more comprehensive and detailed studies are needed to explore the prevalence of myopia in different populations and its associated factors.

Shenyang, the capital of Liaoning Province, China, is the largest city in northeastern China and has a long history of integration of Han, Manchu, Mongolian, Korean ethnic groups, etc. Therefore, the results of the study on myopia in the Shenyang population would be highly representative. The purpose of this study was to investigate the prevalence and potential associated factors of myopia in students from Shenyang, China. The findings will contribute to further understanding of myopia and provide information for the development of effective prevention and treatment strategies.

## 2. Methods

### 2.1. Investigation objects

**Figure 1:**
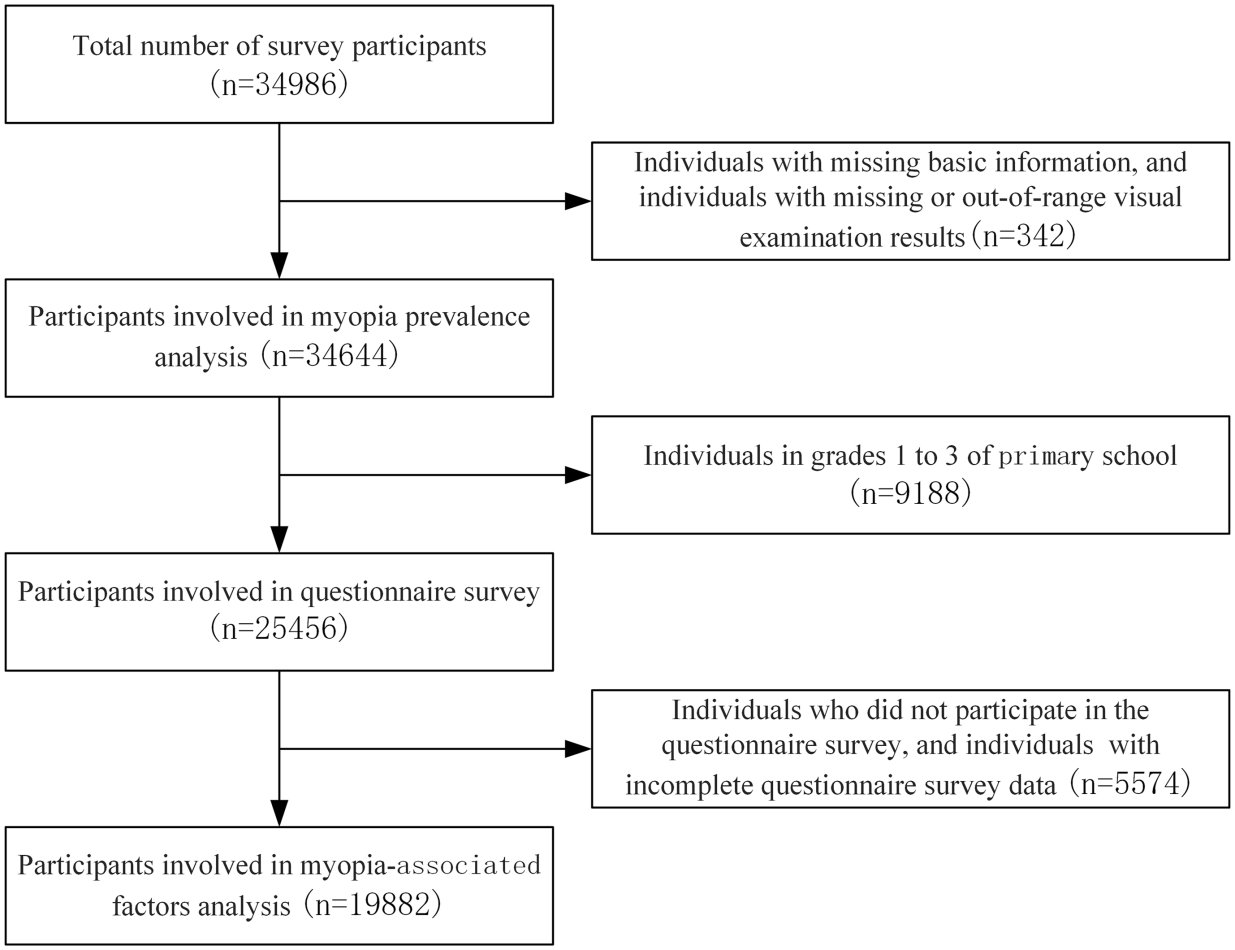
Flow chart for the selection of participants.

The survey was conducted in November–December 2021, using a multi-stage stratified whole-group sampling method. Two primary schools, two middle schools, and two general high schools were selected in each of the five districts in Shenyang (Heping District, Shenhe District, Huanggu District, Dadong District, and Tiexi District), while one vocational high school was selected in the district where there was a vocational high school. For the remaining eight districts and counties (Sujiatun District, Hunnan District, Shenbei District, Yuhong District, Liaocheng District, Xinmin City, Kangping County, and Faku County), two primary schools, two middle schools and one general high school were selected from each district. A total of 73 schools, including 26 primary schools, 26 middle schools, 18 general high schools, and 3 vocational high schools were included in the study. In each grade level of the chosen schools, 2–3 classes (ensuring >100 students in each grade level) were selected as survey subjects. As shown in Figure 1, all survey objects underwent vision examination, while students from grade 4 or above in primary schools participated in the questionnaire survey. In total, 34,644 objects participated in the prevalence of myopia analysis, and 19,882 objects participated in the analysis of myopia-associated factors.

The present investigation was reviewed and approved by the Ethics Committee of the Shenyang Center for Disease Prevention and Control (sycdc-2020-001), and the legal guardians of the participants provided written informed consent to participate in this study.

### 2.2. Investigation methods

#### 2.2.1. Vision examination

The standard logarithmic visual acuity chart and the automatic computerized optometry method under non-ciliary muscle paralysis were used to test the students’ left and right eyes’ naked eye visual acuity. The visual acuity chart used in the study is from GB/T 11533-2011 Standard for logarithmic visual acuity charts, which is a 5-mark record. The test results were recorded by the testing physician on-site in the Liaoning Monitoring Information System for Common Diseases and Health Factors of Students.

#### 2.2.2. Questionnaire survey

The questionnaire was developed by the Chinese Center for Disease Control and Prevention (CDC) and students filled out questionnaires on WeChat. It took approximately 10–15 min to complete the questionnaire. Before completing the questionnaire, the surveyor explained the significance of the survey, emphasized the confidentiality of the questionnaire, and asked students to keep an appropriate distance from each other to ensure students respond seriously. Additionally, any questions that were not understood would be explained by the investigator until the students could understand the questions correctly in order to guarantee the credibility of the results. The survey content was listed in Table 1, which mainly included several aspects such as parents’ myopia, environment of using eyes, habits of using eyes, screen use, outdoor activities, sleep, etc. The results of the questionnaire were uploaded directly to the Liaoning Monitoring Information System for Common Diseases and Health Factors of Students.

**Table 1:** Analysis of associated factors for myopia among primary and secondary school students in Shenyang in 2021.

Variables	Levels	Non-myopia (*N* = 5,446)	Myopia (*N* = 14,436)	OR (univariate analysis)	OR (multivariate analysis)
Sex	Male	3,093 (56.8%)	6,772 (46.9%)		
	Female	2,353 (43.2%)	7,664 (53.1%)	1.49 (1.40–1.58, *p* < 0.001)	1.50 (1.40–1.60, *p* < 0.001)
Educational stage	Primary 4-6th grade	2,946 (54.1%)	4,322 (29.9%)		
	Middle school	1,578 (29.0%)	5,240 (36.3%)	2.26 (2.10–2.44, *p* < 0.001)	2.34 (2.15–2.55, *p* < 0.001)
	General high school	586 (10.8%)	4,102 (28.4%)	4.77 (4.32–5.26, *p* < 0.001)	4.59 (4.07–5.18, *p* < 0.001)
	Vocational high school	336 (6.2%)	772 (5.3%)	1.57 (1.37–1.79, *p* < 0.001)	1.46 (1.24–1.71, *p* < 0.001)
Region	Urban	4,079 (74.9%)	10,673 (73.9%)		
	Rural	1,367 (25.1%)	3,763 (26.1%)	1.05 (0.98–1.13, *p* = 0.165)	
Ethnicity	Han	4,209 (77.3%)	11,161 (77.3%)		
	Others	1,237 (22.7%)	3,275 (22.7%)	1.00 (0.93–1.08, *p* = 0.967)	
Family genetic history	Neither parent is myopic	3,769 (69.2%)	7,740 (53.6%)		
	Father is myopic	514 (9.4%)	2092 (14.5%)	1.98 (1.79–2.20, *p* < 0.001)	2.16 (1.94–2.41, *p* < 0.001)
	Mother is myopic	830 (15.2%)	2,979 (20.6%)	1.75 (1.60–1.91, *p* < 0.001)	2.00 (1.83–2.19, *p* < 0.001)
	Both parents are myopic	333 (6.1%)	1,625 (11.3%)	2.38 (2.10–2.69, *p* < 0.001)	2.94 (2.58–3.35, *p* < 0.001)
Frequency of changing seat	Weekly	3,158 (58.0%)	7,879 (54.6%)		
	Biweekly	785 (14.4%)	2,136 (14.8%)	1.09 (1.00–1.20, *p* = 0.064)	1.00 (0.91–1.10, *p* = 0.968)
	Once a month/no change	1,503 (27.6%)	4,421 (30.6%)	1.18 (1.10–1.27, *p* < 0.001)	0.96 (0.88–1.04, *p* = 0.282)
Adjust the height of the desk and chair according to the height	Non-adjustable	1856 (34.1%)	5,804 (40.2%)		
	Once a year	736 (13.5%)	1794 (12.4%)	0.78 (0.70–0.86, *p* < 0.001)	0.92 (0.83–1.03, *p* = 0.135)
	Once a term	1,569 (28.8%)	3,701 (25.6%)	0.75 (0.70–0.82, *p* < 0.001)	1.00 (0.91–1.09, *p* = 0.928)
	Once every 2–3 months	1,285 (23.6%)	3,137 (21.7%)	0.78 (0.72–0.85, *p* < 0.001)	0.98 (0.89–1.08, *p* = 0.666)
Frequency of daily eye exercises at school	Once	3,518 (64.6%)	9,615 (66.6%)		
	Twice and more	1770 (32.5%)	4,106 (28.4%)	0.85 (0.79–0.91, *p* < 0.001)	0.78 (0.73–0.84, *p* < 0.001)
	No	158 (2.9%)	715 (5.0%)	1.66 (1.39–1.98, *p* < 0.001)	1.13 (0.93–1.38, *p* = 0.230)
Recess activity space	Inside the teaching building	1,236 (22.7%)	4,474 (31.0%)		
	Outdoor	4,210 (77.3%)	9,962 (69.0%)	0.65 (0.61–0.70, *p* < 0.001)	0.85 (0.79–0.93, *p* < 0.001)
Average daily time spent doing homework or reading and writing after school	<1 h	2066 (37.9%)	4,436 (30.7%)		
	1–2 h	2016 (37.0%)	5,663 (39.2%)	1.31 (1.22–1.41, *p* < 0.001)	1.01 (0.94–1.10, *p* = 0.744)
	≥2 h	903 (16.6%)	3,632 (25.2%)	1.87 (1.71–2.05, *p* < 0.001)	1.15 (1.04–1.27, *p* = 0.008)
	No	461 (8.5%)	705 (4.9%)	0.71 (0.63–0.81, *p* < 0.001)	0.82 (0.72–0.94, *p* = 0.005)
Average duration of extracurricular tuition per week	No	2,424 (44.5%)	6,457 (44.7%)		
	<3 h	2,317 (42.5%)	5,883 (40.8%)	0.95 (0.89–1.02, *p* = 0.161)	0.97 (0.91–1.05, *p* = 0.496)
	≥3 h	705 (12.9%)	2096 (14.5%)	1.12 (1.01–1.23, *p* = 0.027)	0.90 (0.81–1.01, *p* = 0.064)
Read and write with your chest more than one punch away from the table	No	2,385 (43.8%)	6,935 (48.0%)		
	Yes	3,061 (56.2%)	7,501 (52.0%)	0.84 (0.79–0.90, *p* < 0.001)	1.10 (1.00–1.20, *p* = 0.052)
Read and write with eyes more than one foot away from the book	No	2,158 (39.6%)	6,781 (47.0%)		
	Yes	3,288 (60.4%)	7,655 (53.0%)	0.74 (0.70–0.79, *p* < 0.001)	0.75 (0.68–0.82, *p* < 0.001)
Read and write with fingers one inch from the tip of the pen	No	1878 (34.5%)	5,425 (37.6%)		
	Yes	3,568 (65.5%)	9,011 (62.4%)	0.87 (0.82–0.93, *p* < 0.001)	1.09 (0.99–1.19, *p* = 0.069)
Average daily computer hours	No	3,104 (57.0%)	8,205 (56.8%)		
	<1 h	1,583 (29.1%)	4,067 (28.2%)	0.97 (0.91–1.04, *p* = 0.434)	
	≥1 h	759 (13.9%)	2,164 (15.0%)	1.08 (0.98–1.18, *p* = 0.109)	
Hours of mobile electronic devices use	No	2,261 (41.5%)	5,542 (38.4%)		
	<0.5 h	836 (15.4%)	2,174 (15.1%)	1.06 (0.97–1.16, *p* = 0.215)	1.15 (0.99–1.33, *p* = 0.076)
	≥0.5 h	2,349 (43.1%)	6,720 (46.6%)	1.12 (1.03–1.22, *p* = 0.005)	1.14 (0.99–1.31, *p* = 0.068)
Reading a book or electronic screen in direct sunlight	No/occasionally	5,192 (95.3%)	13,674 (94.7%)		
	Often	192 (3.5%)	562 (3.9%)	1.11 (0.94–1.31, *p* = 0.215)	
	Always	62 (1.1%)	200 (1.4%)	1.22 (0.92–1.63, *p* = 0.166)	
Reading the electronic screen with the lights off after dark	No/occasionally	5,077 (93.2%)	13,095 (90.7%)		
	Often	247 (4.5%)	925 (6.4%)	1.45 (1.26–1.68, *p* < 0.001)	1.08 (0.91–1.28, *p* = 0.378)
	Always	122 (2.2%)	416 (2.9%)	1.32 (1.08–1.62, *p* = 0.007)	1.11 (0.88–1.42, *p* = 0.378)
Reading a book or electronic screen while lying down or lying on the back	No/occasionally	4,694 (86.2%)	11,866 (82.2%)		
	Often	626 (11.5%)	2,157 (14.9%)	1.36 (1.24–1.50, *p* < 0.001)	1.09 (0.98–1.22, *p* = 0.128)
	Always	126 (2.3%)	413 (2.9%)	1.30 (1.06–1.59, *p* = 0.012)	0.89 (0.69–1.16, *p* = 0.394)
Reading a book or electronic screen while walking or riding in a car	No/occasionally	5,177 (95.1%)	13,372 (92.6%)		
	Often	219 (4.0%)	842 (5.8%)	1.49 (1.28–1.73, *p* < 0.001)	0.84 (0.70–1.00, *p* = 0.052)
	Always	50 (0.9%)	222 (1.5%)	1.72 (1.26–2.34, *p* < 0.001)	1.27 (0.86–1.86, *p* = 0.226)
When using the computer, eyes from the screen more than 66 cm	No/occasionally	1,620 (29.7%)	4,801 (33.3%)		
	Often/always	3,826 (70.3%)	9,635 (66.7%)	0.85 (0.79–0.91, *p* < 0.001)	0.97 (0.90–1.05, *p* = 0.408)
When using eyes at close range, how often to rest your eyes	< 15 min	1,405 (25.8%)	3,355 (23.2%)		
	≤ 15 < 30 min	1718 (31.5%)	4,649 (32.2%)	1.13 (1.04–1.23, *p* = 0.003)	1.03 (0.94–1.13, *p* = 0.517)
	≤ 30 < 60 min	835 (15.3%)	2,669 (18.5%)	1.34 (1.21–1.48, *p* < 0.001)	1.06 (0.96–1.18, *p* = 0.254)
	≥ 60 min	1,488 (27.3%)	3,763 (26.1%)	1.06 (0.97–1.15, *p* = 0.194)	0.94 (0.86–1.04, *p* = 0.229)
Daytime outdoor activity hours	< 1 h	1,295 (23.8%)	4,088 (28.3%)		
	1–2 h	2,293 (42.1%)	5,942 (41.2%)	0.82 (0.76–0.89, *p* < 0.001)	0.92 (0.85–1.01, *p* = 0.076)
	≥ 2 h	1858 (34.1%)	4,406 (30.5%)	0.75 (0.69–0.82, *p* < 0.001)	0.92 (0.84–1.01, *p* = 0.068)
Average daily sleep duration	< 8 h	1,596 (29.3%)	6,739 (46.7%)		
	≥ 8 h	3,850 (70.7%)	7,697 (53.3%)	0.47 (0.44–0.51, *p* < 0.001)	0.90 (0.83–0.98, *p* = 0.018)

### 2.3. Myopia evaluation criteria

The criteria for determining myopia were that the standard logarithmic visual acuity of the naked eye was below 5.0 and the spherical equivalent by computerized optometry was below −0.50D under non-ciliary muscle paralysis. Anyone who was determined to be myopic in one eye or confirmed to be wearing an orthokeratology lens would be counted in the total number of myopia. In this study, low myopia, moderate myopia and high myopia were defined as monocular standard logarithmic visual acuity <5.0 and −3.0D ≤ spherical equivalent < −0.5D, monocular standard logarithmic visual acuity <5.0 and −6.0D ≤ spherical equivalent < −3.0D, and monocular standard logarithmic visual acuity <5.0 and spherical equivalent < −6.0D, respectively.

### 2.4. Quality control

The testing staff used uniform testing methods and uniform equipment to conduct the survey. The testing team consisted of a testing captain, at least one ophthalmologist who had a national license related to optometry, and several professionals who were certified as optometry-related technologists or nurses, or professionals in the field of school health. All inspectors were required to receive unified training, be proficient in testing methods and qualified by the examination before starting the detection work.

During the survey, retesting subjects were randomly selected according to 5% of the number of students tested on that day, and retesting of left and right eye visual acuity, visual acuity with lenses, spherical and cylindrical lenses was conducted. Those who wore orthokeratology lens were not included in the vision retesting detection. The error of the naked eye and the vision with glasses exceeds ±1 line and the error of spherical equivalent exceeds ±0.50D, which was regarded as the detection error. If the incidence of error was greater than 5%, the detection team should promptly convene a meeting to investigate the causes and improve the approach, while reviewing, retesting and correcting indicators that exceed the allowable error range. In case the incidence of error was greater than 10%, it was determined that all the testing data for the day was invalid and must be retested.

### 2.5. Data analysis

EpiData 3.1 was used to create the database for parallel double entry, and R 4.2.3 software was employed for statistical analysis. The measurement data were described as mean ± standard deviation or median (quartiles), and the count data were described as number of cases (percentage). The χ^2^ test was adopted for univariate analysis. Unconditional dichotomous logistic regression was employed for multivariate analysis, where variables with *p* < 0.1 in the univariate analysis were selected as independent variables, and myopia was considered as the dependent variable. Dose–response analyses were performed using restricted cubic splines for the prevalence of myopia in relation to sleep duration and hours of mobile electronic device use. Sensitivity analysis of the results was conducted by adjusting the reference levels of some of the indicators, and subgroup analysis was performed for gender and educational stage. The test level was *α* = 0.05.

## 3. Results

### 3.1. Basic information of the survey subjects

As can be seen from Table 2, a total of 34,644 students were investigated, with a median age of 11.9 years and a close ratio of 51% and 49% of males and females, respectively. Primary school students, middle school students, general high school students, and vocational high school students accounted for 53%, 25%, 18%, and 4%, respectively. The students surveyed were predominantly Han Chinese, accounting for 81%, and mainly from urban districts, accounting for 71%. There was a significant difference between the non-myopia group and the myopic group in terms of age, gender and educational stage (*p* < 0.001).

**Table 2:** Basic characteristics of survey subjects.

Variables	Total, *N* = 34,644 (100%)^a^	Non-myopia, *N* = 13,895 (40%)^a^	Myopia, *N* = 20,749 (60%)^a^	*p*-value^b^
Age (years)	11.9 (9.0, 14.6)	9.3 (7.6, 12.0)	13.3 (11.0, 15.6)	<0.001
Sex				<0.001
Male	17,563 (51%)	7,604 (55%)	9,959 (48%)	
Female	17,081 (49%)	6,291 (45%)	10,790 (52%)	
Educational stage				<0.001
Primary school	18,392 (53%)	10,619 (77%)	7,773 (37%)	
Middle school	8,737 (25%)	2,136 (15%)	6,601 (32%)	
General high school	6,151 (18%)	742 (5%)	5,409 (26%)	
Vocational high school	1,364 (4%)	398 (3%)	966 (5%)	
Ethnicity				0.078
Han	27,983 (81%)	11,160 (80%)	16,823 (81%)	
Others	6,661 (19%)	2,735 (20%)	3,926 (19%)	
Region^c^				0.6
Urban	24,757 (71%)	9,950 (72%)	14,807 (71%)	
Rural	9,887 (29%)	3,945 (28%)	5,942 (29%)	

a Median (IQR); *n* (%).

b Wilcoxon rank sum test; Pearson’s Chi-squared test.

c Urban area refers to Heping District, Shenhe District, Huangg District, Dandong District, Tiexi District, Hunnan District, Shenyang North District; Rural area refers to Sujiatun District, Yuhong District, Liaozhong District, Xinmin City, Kangping County, Faku County.

### 3.2. Prevalence of myopia among primary and secondary school students

**Figure 2:**
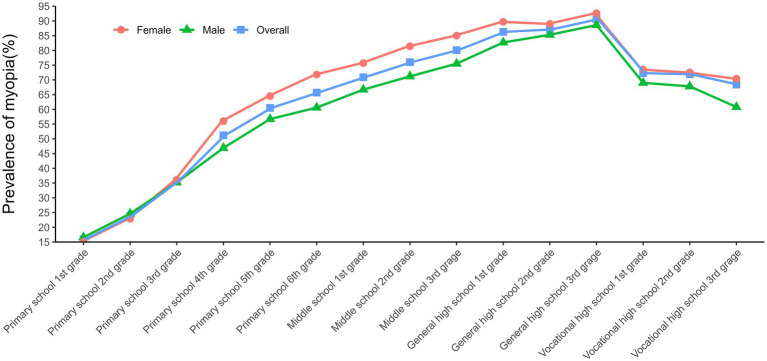
The prevalence of myopia in different grade.

As shown in Table 3, the overall prevalence of myopia among primary and secondary school students in Shenyang was 60% in 2021. The prevalence of mild myopia was 45%, accounting for 75.3% of all myopia, while the prevalence of moderate myopia and high myopia was 13% and 1.9%, accounting for 21.5% and 3.2% of all myopia, respectively. Moreover, the prevalence of myopia among female students was 63%, which was higher than the prevalence of 57% among male students. The prevalence of myopia generally increased with educational stage and grade level except for vocational high school (Figure 2; Supplementary Table S1). In addition, the prevalence of myopia was similar among different ethnic groups, as well as in urban and rural areas except for high myopia.

**Table 3:** Prevalence of myopia among primary and secondary school students in Shenyang in 2021.

Total *N* = 34,644	Total myopia *N* = 20,749 (60%)^a^	Low myopia *N* = 15,630 (45%)^a^	Moderate myopia *N* = 4,452 (13%)^a^	High myopia *N* = 667 (1.9%)^a^	*p*-value^b^
Sex					<0.001
Male (17,563)	9,959 (57%)	7,610 (43%)	2,040 (12%)	309 (1.8%)	
Female (17,081)	10,790 (63%)	8,020 (47%)	2,412 (14%)	358 (2.1%)	
Educational stage					<0.001
Primary school (18,392)	7,773 (42%)	6,937 (38%)	793 (4.3%)	43 (0.2%)	
Middle school (8,737)	6,601 (76%)	4,886 (56%)	1,539 (18%)	176 (2.0%)	
General high school (6,151)	5,409 (88%)	3,130 (51%)	1,893 (31%)	386 (6.3%)	
Vocational high school (1,364)	966 (71%)	677 (50%)	227 (17%)	62 (4.5%)	
Ethnicity					0.20
Han (27,983)	16,823 (60%)	12,653 (45%)	3,620 (13%)	550 (2.0%)	
Others (6,661)	3,926 (59%)	2,977 (45%)	832 (12%)	117 (1.8%)	
Region					<0.001
Urban (24,757)	14,807 (60%)	11,086 (45%)	3,189 (13%)	532 (2.1%)	
Rural (9,887)	5,942 (60%)	4,544 (46%)	1,263 (13%)	135 (1.4%)	

a *n* (%).

b Pearson’s Chi-squared test.

### 3.3. Dose–response analysis

To further investigate the key factors affecting myopia, the effect of associated factors on myopia was analyzed using restricted cubic splines. The results indicated a non-linear relationship between sleep duration and myopia risk (*p* < 0.0001), whereas there was no nonlinear relationship between the length of mobile electronic device use and myopia risk (*p* = 0.8428).

**Figure 3:**
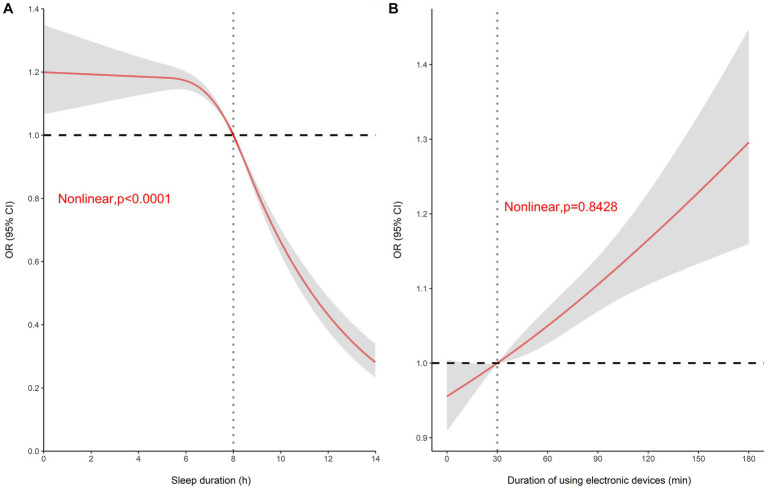
Restricted cubic spline analysis. **(A)** Dose–response relationship between sleep duration and myopia risk (OR). **(B)** Dose–response relationship between hours of mobile electronic device use and myopia risk (OR).

With regard to sleep duration, less than 8 h of sleep was associated with an increased risk of myopia, whereas more than 8 h of sleep was a protective factor for myopia, and the longer the sleep duration, the stronger the effect (Figure 3A). Conversely, the risk of myopia increased with the duration of mobile electronic device use. Interestingly, the length of mobile device use was significantly associated with the increased risk of myopia (*p* < 0.05) when exceeded 30 min (Figure 3B). Therefore, sleep duration was categorized as <8 h and ≥ 8 h, and the length of mobile electronic device use was categorized as no use, <0.5 h, and ≥ 0.5 h according to the above results in the subsequent associated factor analysis.

### 3.4. Analysis of associated factors affecting myopia

As shown in Table 1; Supplementary Figure S1, univariate logistic regression indicated that associated factors for myopia development involved female students, high school grades, family genetic history of myopia, low frequency of changing seats, doing homework or reading and writing after school for ≥1 h, spending ≥3 h per week on extracurricular tutoring, using mobile electronic devices for a long time, reading the electronic screen with the lights off after dark, reading a book or electronic screen while lying down or lying on the back, reading a book or electronic screen while walking or riding in a car, and using your eyes for long hours at close range without rest. Correspondingly, regular adjustment of desk and chair height according to height, doing eye exercises twice a day or more, going outdoors at recess, reading and writing with your chest more than one fist from the table, reading and writing with eyes more than one foot from books, reading and writing with fingers one inch from the tip of the pen, using a computer with eyes over 66 cm from the screen, outdoor activity ≥1 h during the day, and sleep ≥8 h per day were associated factors for preventing myopia. What’s more, the final multivariate analysis demonstrated that female students, high school grades, family genetic history, and doing homework or reading and writing for ≥2 h after school were closely associated with the occurrence of myopia, while doing eye exercises twice a day or more, going outdoors during recess, reading and writing with eyes more than one foot from a book, and sleeping duration ≥8 h per day were identified as associated factors for preventing myopia (Table 1; Supplementary Figure S1).

### 3.5. Subgroup analysis of associated factors affecting myopia

**Figure 4:**
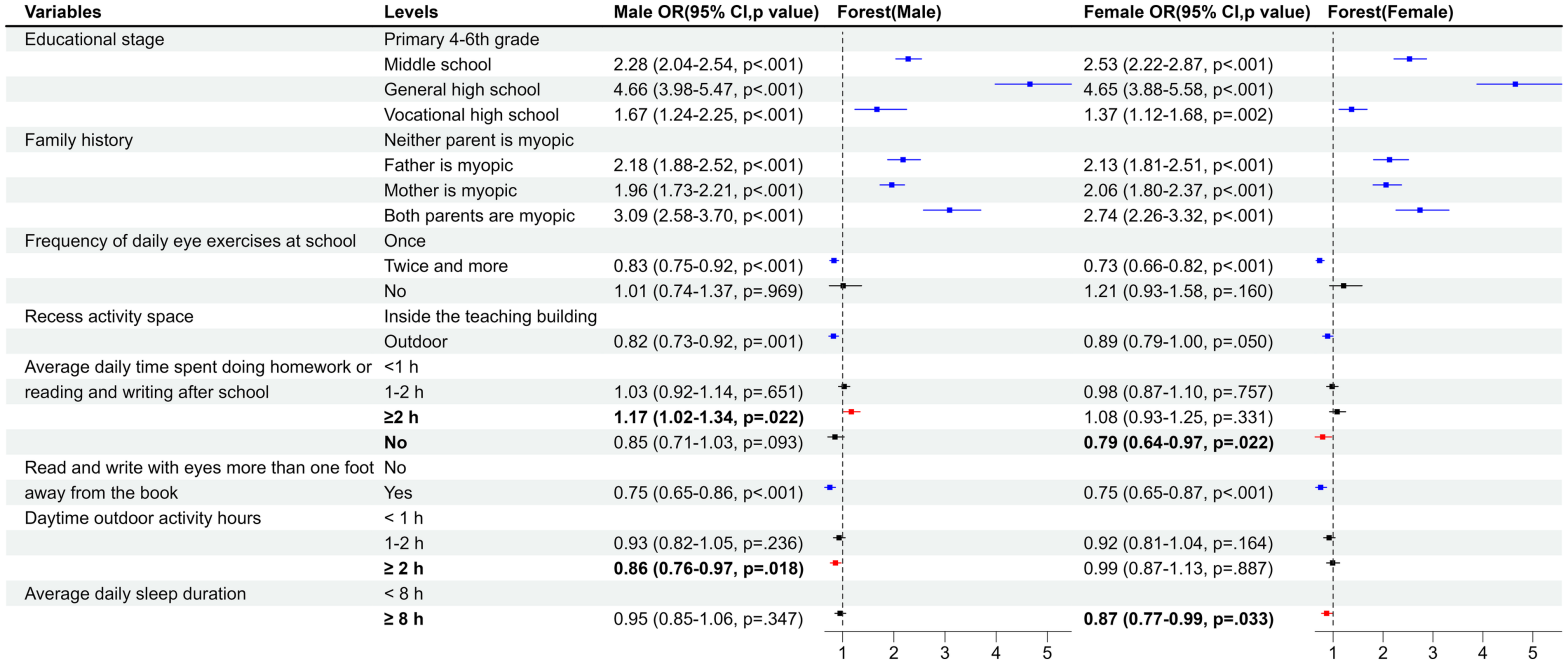
The forest plot for subgroup in gender.

For the gender subgroup analysis, the results suggested that the factors influencing myopia were not exactly the same between males and females (Figure 4; Supplementary Tables S2, S3). Specifically, ≥2 h of outdoor activity during the day was identified as an associated factor for preventing myopia in boys, but not seen in girls. In contrast, ≥8 h of sleep was identified as an associated factor for preventing myopia in girls, but not seen in boys. Additionally, ≥2 h of homework or reading and writing after school was associated with a higher risk of myopia in boys compared with <1 h of homework or reading and writing after school, while girls showed no homework as an associated factor for preventing myopia.

**Figure 5:**
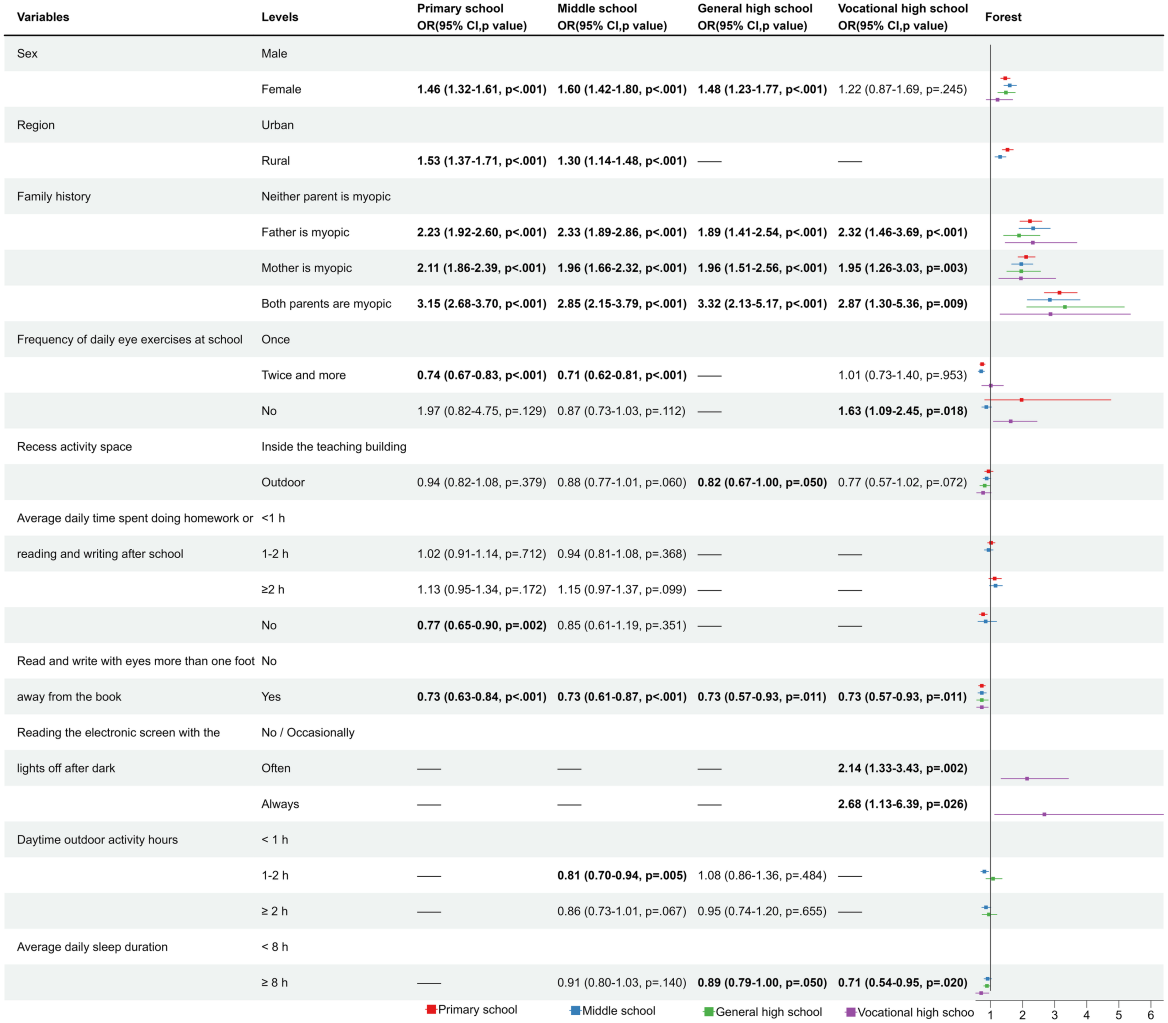
The forest plot for subgroup in educational stage. — indicates that the variable had a *p*-value greater than 0.1 in the univariate analysis, therefore it was not included in the multivariate analysis. Bold font indicates significant results (*p* < 0.05).

As shown in Figure 5; Supplementary Tables S4–S7, subgroup analysis by educational stage revealed that in primary and middle school stages, the risk of myopia is higher in rural areas compared to urban areas. Interestingly, compared to doing eye exercises once a day, doing eye exercises twice a day was beneficial in preventing myopia, except at the high school level. Adequate sleep duration (≥8 h) was found to be associated with a lower risk of myopia only in the high school stage, while no such association was observed in the primary and middle school stages. Myopia rates were lower among elementary school students who did not do homework after school, middle school students who engaged in 1–2 h of outdoor activity per day, and general high school students who took outdoor breaks between classes. However, vocational high school students who did not perform daily eye exercises and engaged in reading electronic screens with the lights off after dark were more likely to develop myopia. These observations were not observed in other subgroups.

### 3.6. Sensitivity analysis

To verify the stability of the results, we adjusted the length of time using mobile electronic devices from three levels of no use, <0.5 h, and ≥ 0.5 h to two levels of use and no use, and adjusted the reference level for the length of time doing homework or reading and writing after school each day from <1 h to no homework for sensitivity analysis. The findings of multivariable analysis did not change (Supplementary Table S8), indicating that the regression model was more stable.

## 4. Discussion

In recent years, the prevalence of myopia among students across China is generally high and showing an increasing trend, whereas the age of patients is getting younger and younger. The current survey found that the myopia detection rate among students in Shenyang was 60% in 2021, which is higher than the domestic average of 52.7% (18). The prevalence of myopia was much higher in the Chinese population compared to students in other countries. The Irish Eye Study surveyed 1,626 participants, and the prevalence of myopia was 3.3 and 19.9% in participants aged 6–7 and 12–13 years, respectively (19). An investigation in Germany showed little change in the prevalence of myopia in German children and adolescents over a period of approximately 10 years, with a prevalence of myopia of approximately 11.6% in 0–17 years of age (20). Studies in Australia (21), Singapore (22), and Taiwan (23) have indicated that the prevalence of myopia is typically high among children of Chinese descent, suggesting a genetic susceptibility to myopia among the Chinese population. Liu et al. (24) and Li et al. (25) have also confirmed the existence of susceptibility genes for myopia. However, Rose et al. (26) reported that compared to Chinese children living in Singapore, Chinese children growing up in Sydney had a lower prevalence of myopia, which is believed to be associated with an increase in outdoor activity time. Therefore, the high prevalence of myopia among Chinese students may be attributed to the interaction between genetic susceptibility and environmental factors such as excessive academic workload and inadequate outdoor activities. The Chinese government needs to take further measures to reduce the prevalence of myopia.

This study demonstrated that the prevalence of myopia increased with grade level, with the highest prevalence of 88% in general high school, which was related to the accumulation of myopia, but also to the increasing study tasks and more frequent use of eyes as the grade level increased. This can be confirmed by the difference in the prevalence of myopia between general high schools and vocational high schools, which are of the same grade level. This is probably because vocational high schools are technical schools where students do not have to take the college entrance exams and are under relatively less pressure to study. Moreover, students in vocational high schools do not work as hard as students in general high schools. In short, this implies that students in vocational high schools used their eyes significantly less than students in general high schools, so that myopia prevalence in vocational high schools (71%) was lower than in regular high schools and even lower than in middle school (76%). It was evident that students’ learning tasks were closely related to the occurrence of myopia, which was also consistent with the statistical findings that the risk of myopia among students with no homework after school was 0.82 times higher (95% CI = 0.72–0.94) than that of students with less than 1 h of homework, while the risk of myopia among students with more than 2 h of homework was 1.15 (1.04–1.27) than that of students with less than 1 h of homework. In recent years, although various localities in China have introduced some policies to reduce the burden, and schools are actively implementing them, the burden of students’ schoolwork is still very heavy (27, 28). The “starting line” mentality of parents, transferring the burden reduced from schools onto their children, has led to a significant increase in eye strain for students (29). As a result, the prevalence of myopia among children remains high, which should draw the attention of parents.

Similar to other chronic diseases, the development of myopia is mainly influenced by genetic and external environmental factors. Parental history of myopia was found to be associated with the development of myopia as early as 1989 (30). Studies from different countries have come to a common conclusion that parental myopia is the most important factor related to the development of myopia in children (31, 32). In this study, students with only a father’s myopia, only a mother’s myopia, and both parents’ myopia had 2.16, 2.00, and 2.94 times higher risk of developing myopia, respectively, compared to students whose parents were not myopia. This indicates that students with parental myopia have a higher risk of developing myopia. Since genetic factors are irresistible, students with a family history of myopia should pay more attention to eye hygiene.

There are multiple external factors that can influence the development of myopia, such as the height of desks and chairs, eye usage habits, reading and writing posture, use of electronic products, and sleep duration. Different surveys yield varying results regarding their impact on myopia occurrence (33). The results of the multivariate logistic regression of this survey indicated that doing eye exercises twice a day or more at school, being outdoors during recess, reading and writing with eyes more than one foot away from books, and sleeping for more than 8 h could prevent the occurrence of myopia. Previous studies have shown that eye exercises can relieve eye muscle tension and make the eyes fully relaxed, as well as improve eye blood circulation and further promote eye health, which can effectively reduce the risk of myopia (34, 35). Therefore, schools should supervise students to complete eye exercises with high quality rather than as a formality.

Being outdoors during recess may prevent myopia, probably because physical activity can promote blood circulation and supply within the eye, thereby inhibiting excessive elongation of the eyeball and reducing the occurrence of myopia in children and adolescents (36). It has been suggested that the mechanism of the protective effect of being outdoors on myopia involves the photostimulation of retinal dopamine release, as an increase in dopamine release appears to inhibit the increase in axial extension, which is the structural basis of myopia (37). However, some studies concluded that there was no correlation between physical activity and myopia in children and adolescents, whereas the protective effect of outdoor physical activity on the vision of children and adolescents was mainly attributed to outdoor factors. For example, outdoor activities promote the production of vitamin D in the body, and high levels of vitamin D are beneficial in preventing the occurrence of myopia (38). Regardless of the underlying mechanisms of its occurrence, outdoor activities during recess are important in preventing the occurrence of myopia in students. Therefore, schools should establish certain systems to ensure that every student has to participate in outdoor activities during break time.

When the reading and writing posture is incorrect and the eyes are too close to the books, the eyes need to adjust the distance frequently so that the eye muscles are under tension for a long time, which will easily cause the occurrence of myopia (13). Therefore, maintaining a good reading and writing posture, keeping the eyes more than one foot away from books, is beneficial in preventing the onset of myopia. Parents and school teachers should pay attention to timely correction of students’ poor reading and writing posture.

The results of the relationship between insufficient sleep and myopia are inconsistent. Liu et al. (16) conducted a cross-sectional survey of 13,642 students aged 9–18 years in China and found that sleep duration <8 h per day was associated with decreased visual acuity. Lin et al. (39) investigated the relationship between myopia prevalence and sleep in 9,530 Chinese school-age children and demonstrated that the prevalence of myopia gradually decreased with increasing sleep duration and that sleep duration was negatively associated with the risk of myopia in a multivariate analysis. In contrast, however, Li et al. (15), in a cross-sectional study, did not find an association between sleep quality, duration and myopia in primary school children from Singapore. Huang et al. (17) reported the relationship between sleep duration and myopia in Chinese children during the COVID-19 Epidemic, and a total of 1,140 children were included in the analysis, which showed that sleep duration was not associated with myopia after adjusting for age, gender, parental myopia, outdoor hours, and hours of continuous uninterrupted near work. The dose–response results of this survey indicated a non-linear relationship between sleep duration and the risk of myopia, with a threshold point of 8 h. The results of the multivariate logistic regression analysis ultimately showed that sleep duration below 8 h was associated with an increased risk of myopia. Interestingly, the sex-stratified analysis showed that this phenomenon was only seen in girls but not in boys, which was similar to that reported by Wei et al. (40), who reported that sleep duration in girls was associated with myopia progression and eye axis lengthening in a 4-year follow-up study of Chinese children, while no such association was found in boys. Wei suggested that this phenomenon may be related to the earlier onset of puberty in girls. It is evident that the effect of sleep on myopia may be influenced by sex factors.

Currently, although the reports on the influence of sex on myopia are not entirely consistent, overall, the prevalence of myopia is higher in females than in males (41–43). Our study showed a higher prevalence of myopia in girls than in boys (63% vs. 57%), and the risk of myopia was 1.50 times higher in girls than in boys (95% CI = 1.40–1.60), which was the same as most reported results. Some researchers suggested that myopia may be related to hormonal changes during puberty, as high levels of estrogen during adolescence may change the shape of the eye and lead to myopia (44), whereas others proposed that sex differences in myopia were related to girls being less active outdoors, but spending more time reading and writing than boys (45–47). Our current findings of gender stratified analysis were consistent with the above findings. In terms of duration of outdoor activity during the daytime, univariate analysis of the total population indicated that 1–2 h and ≥ 2 h of outdoor activity were significant protective factors, with ORs of 0.82 (95% CI = 0.76–0.89, *p* < 0.001) and 0.75 (95% CI = 0.69–0.82, *p* < 0.001), respectively. However, in the multivariable analysis, ORs were 0.92 (95% CI = 0.85–1.01, *p* = 0.076) and 0.92 (95% CI = 0.84–1.01, *p* = 0.068) respectively, showing no statistical significance, but the *p*-values are close to 0.05. Gender-stratified analysis revealed that outdoor activity for more than 2 h was a protective factor against myopia in boys, with an OR of 0.86 (95% CI = 0.76–0.97, *p* = 0.018), However, for girls, the OR was 0.99 (0.87–1.13, *p* = 0.887), indicating no statistical significance. The lack of significance in girls may be related to the fact that girls were less active outdoors, which consequently influenced the overall population not exhibiting significance. In terms of the duration of doing homework or reading and writing after school, compared to not having any homework, spending less than 1 h on homework or reading and writing was associated with an increased risk of myopia for girls, which indicated that girls were more susceptible to the influence of duration of homework or reading and writing. Therefore, girls should pay more attention to eye hygiene and try to engage in outdoor activities as much as possible.

In the subgroup analysis by educational stage, some interesting results were obtained. We initially hypothesized that urban children would have a higher risk of myopia than rural children due to higher academic stress (48). However, children in rural areas had a higher risk of myopia than urban children, which was contrary to our expectations. It is hypothesized that this may be related to lower expectations from parents in rural areas and easier access for children to electronic devices. Moreover, the protective effect of eye exercises against myopia was mainly observed in elementary and middle school students, suggesting that eye exercises may have a stronger beneficial effect on developing eyes, as well as the importance of reinforcing eye exercises for elementary and middle school students. We also found that among vocational school students, reading electronic screens with lights off after dark was associated with an increased risk of myopia, which may be due to a lack of strict management for vocational school students, resulting in the possibility that some students may continue to use electronic devices even after lights out. This finding implied that reading electronic screens with lights off after dark can also contribute to the development of myopia. In addition, the preventive effects of having ≥8 h of sleep and being in outdoor activities during recess against myopia were observed specifically in high school students. This may be attributed to the longer duration of sleep and outdoor activities during breaks among elementary and middle school students, which could mask the significance of these factors (15).

Although many epidemiologic studies have determined the prevalence and incidence of myopia in Asia, our study has some strengths. First, it is part of a nationwide monitoring and intervention project for common diseases and health factors among students in China. The uniformity of methodology, strict quality control measures of the study, professionalism of the researchers, and large sample size make this study more reflective of the myopia status of the Chinese student population. Moreover, the study comprehensively examined the factors influencing myopia and uncovered findings not observed in other studies, such as the effectiveness of outdoor activities during breaks in preventing myopia. In addition, a more comprehensive and detailed analysis of the data highlights the need for greater attention to myopia among rural primary and middle school students in China, the effectiveness of eye exercises for younger students, and the importance of sleep duration and outdoor activities for girls. However, despite the large sample size of this study, this was a cross-sectional investigation and we were unable to draw conclusions regarding the causality of myopia occurrence. Therefore, a cohort study is needed to validate our findings. In addition, the factors influencing different levels of myopia were not explored further in the current study, which led to the possibility of missing some important information.

In summary, the prevalence of myopia among primary and secondary school students in Shenyang is high. Apart from genetic factors, the main contributing factors to this high prevalence of myopia are heavy study burden and excessive eye use of students. Moreover, reading and writing with eyes too close to the book and lack of sleep can increase the risk of myopia, while doing more eye exercises and going outdoors would help reduce the risk of myopia. These findings not only provide a general impression of the prevalence of myopia in Shenyang, but also have important implications for the prevention and control of myopia in northeastern China.

## Data availability statement

The original contributions presented in the study are included in the article/Supplementary material, further inquiries can be directed to the corresponding authors.

## Ethics statement

The studies involving humans were approved by Ethics Committee of the Shenyang Center for Disease Control and Prevention. The studies were conducted in accordance with the local legislation and institutional requirements. Written informed consent for participation in this study was provided by the participants’ legal guardians/next of kin. Written informed consent was obtained from the individual(s), and minor(s)’ legal guardian/next of kin, for the publication of any potentially identifiable images or data included in this article.

## Author contributions

DZ, BS, MW, LZ, and LG: conceptualization and writing—review and editing. DZ, BS, MW, and HL: methodology. HL and LZ: software. BS and HL: validation. DZ, LZ, and LG: formal analysis, writing—original draft preparation, and visualization. DZ, BS, LZ, and LG: data curation. MW, LZ, and LG: supervision. BS, MW, and LG.: project administration. All authors contributed to the article and approved the submitted version.

## Funding

This research was supported by Liaoning Science and Technology Plan Project (No. 2022JH1/10800071).

## Conflict of interest

The authors declare that the research was conducted in the absence of any commercial or financial relationships that could be construed as a potential conflict of interest.

## Publisher’s note

All claims expressed in this article are solely those of the authors and do not necessarily represent those of their affiliated organizations, or those of the publisher, the editors and the reviewers. Any product that may be evaluated in this article, or claim that may be made by its manufacturer, is not guaranteed or endorsed by the publisher.
