# Supplementary material for: Prevalence and associated factors of myopia among school students in Shenyang, China: a cross-sectional study
Source: Front Public Health. 2023 Aug 30;11:1239158. doi: 10.3389/fpubh.2023.1239158 (PMC10499391; doi:10.3389/fpubh.2023.1239158)
Supplement: Supplementary file 1 [file Table_1.doc]

**Supplementary Tables and Figure**

| **Supplementary Table1** **Prevalence of myopia at different grade** | | | | | | |
| --- | --- | --- | --- | --- | --- | --- |
| Age (year) | **Overall** | | **Male** | | **Female** | |
|  | Non-myopia,  N = 13,895^1^ | Myopia,  N = 20,749^1^ | Non-myopia,  N = 7,604^1^ | Myopia,  N = 9,959^1^ | Non-myopia,  N = 6,291^1^ | Myopia,  N = 10,790^1^ |
| Primary school 1st grade | 2,527 (84.1%) | 476 (15.9%) | 1,290 (83.4%) | 256 (16.6%) | 1,237 (84.9%) | 220 (15.1%) |
| Primary school 2nd grade | 2,328 (76.2%) | 727 (23.8%) | 1,175 (75.4%) | 384 (24.6%) | 1,153 (77.1%) | 343 (22.9%) |
| Primary school 3rd grade | 2,015 (64.4%) | 1,115 (35.6%) | 1,073 (64.8%) | 582 (35.2%) | 942 (63.9%) | 533 (36.1%) |
| Primary school 4th grade | 1,475 (48.8%) | 1,550 (51.2%) | 837 (53.1%) | 739 (46.9%) | 638 (44.0%) | 811 (56.0%) |
| Primary school 5th grade | 1,175 (39.5%) | 1,798 (60.5%) | 668 (43.3%) | 876 (56.7%) | 507 (35.5%) | 922 (64.5%) |
| Primary school 6th grade | 1,099 (34.3%) | 2,107 (65.7%) | 685 (39.4%) | 1,055 (60.6%) | 414 (28.2%) | 1,052 (71.8%) |
| Middle school 1st grade | 879 (29.1%) | 2,137 (70.9%) | 540 (33.3%) | 1,081 (66.7%) | 339 (24.3%) | 1,056 (75.7%) |
| Middle school 2nd grade | 694 (24.0%) | 2,201 (76.0%) | 438 (28.8%) | 1,081 (71.2%) | 256 (18.6%) | 1,120 (81.4%) |
| Middle school 3rd grade | 563 (19.9%) | 2,263 (80.1%) | 360 (24.5%) | 1,109 (75.5%) | 203 (15.0%) | 1,154 (85.0%) |
| General high school 1st grade | 298 (13.7%) | 1,872 (86.3%) | 184 (17.3%) | 879 (82.7%) | 114 (10.3%) | 993 (89.7%) |
| General high school 2nd grade | 258 (12.9%) | 1,748 (87.1%) | 148 (14.7%) | 860 (85.3%) | 110 (11.0%) | 888 (89.0%) |
| General high school 3rd grade | 186 (9.4%) | 1,789 (90.6%) | 113 (11.4%) | 878 (88.6%) | 73 (7.4%) | 911 (92.6%) |
| Vocational high school 1st grade | 122 (27.7%) | 319 (72.3%) | 36 (31.0%) | 80 (69.0%) | 86 (26.5%) | 239 (73.5%) |
| Vocational high school 2nd grade | 128 (28.1%) | 327 (71.9%) | 19 (32.2%) | 40 (67.8%) | 109 (27.5%) | 287 (72.5%) |
| Vocational high school 3rd grade | 148 (31.6%) | 320 (68.4%) | 38 (39.2%) | 59 (60.8%) | 110 (29.6%) | 261 (70.4%) |
| ^1^n (%) | | | | | | |

**Supplementary Table 2** **Analysis of associated factors for myopia among primary and secondary school students in Shenyang in 2021(****subgroup analysis-Male)**

| **Variables** | **Levels** | **Non-myopia**  (N=3093) | **Myopia**  (N=6772) | OR **(****univariate)** | OR**(****multivariate)** |
| --- | --- | --- | --- | --- | --- |
| **Educational stage** | Primary 4-6th grade | 1693 (54.7%) | 2085 (30.8%) |  |  |
|  | Middle school | 972 (31.4%) | 2575 (38%) | **2.15 (1.95-2.37, p<.001)** | **2.28 (2.04-2.54, p<.001)** |
|  | General high school | 344 (11.1%) | 1963 (29%) | **4.63 (4.06-5.28, p<.001)** | **4.66 (3.98-5.47, p<.001)** |
|  | Vocational high school | 84 (2.7%) | 149 (2.2%) | **1.44 (1.09-1.90, p=.009)** | **1.67 (1.24-2.25, p<.001)** |
| **Region** | Urban | 2292 (74.1%) | 4959 (73.2%) |  |  |
|  | Rural | 801 (25.9%) | 1813 (26.8%) | 1.05 (0.95-1.15, p=.361) |  |
| **Ethnicity** | Han | 2419 (78.2%) | 5229 (77.2%) |  |  |
|  | Others | 674 (21.8%) | 1543 (22.8%) | 1.06 (0.96-1.17, p=.273) |  |
| **Family genetic history** | Neither parent is myopic | 2120 (68.5%) | 3579 (52.8%) |  |  |
|  | Father is myopic | 295 (9.5%) | 1009 (14.9%) | **2.03 (1.76-2.33, p<.001)** | **2.18 (1.88-2.52, p<.001)** |
|  | Mother is myopic | 496 (16%) | 1426 (21.1%) | **1.70 (1.52-1.91, p<.001)** | **1.96 (1.73-2.21, p<.001)** |
|  | Both parents are myopic | 182 (5.9%) | 758 (11.2%) | **2.47 (2.08-2.93, p<.001)** | **3.09 (2.58-3.70, p<.001)** |
| **Frequency of changing seat** | Weekly | 1827 (59.1%) | 3854 (56.9%) |  |  |
|  | Biweekly | 433 (14%) | 964 (14.2%) | 1.06 (0.93-1.20, p=.403) | 0.97 (0.85-1.11, p=.634) |
|  | Once a month/no change | 833 (26.9%) | 1954 (28.9%) | 1.11 (1.01-1.23, p=.034) | 0.92 (0.82-1.03, p=.136) |
| **Adjust the height of the desk and chair according to the height** | Never or non-adjustable | 1010 (32.7%) | 2607 (38.5%) |  |  |
|  | Once a year | 413 (13.4%) | 804 (11.9%) | 0.75 (0.66-0.87, p<.001) | 0.88 (0.76-1.03, p=.105) |
|  | Once a term | 894 (28.9%) | 1739 (25.7%) | 0.75 (0.68-0.84, p<.001) | 0.98 (0.87-1.10, p=.753) |
|  | Once every 2 to 3 months | 776 (25.1%) | 1622 (24%) | 0.81 (0.72-0.91, p<.001) | 0.98 (0.87-1.11, p=.778) |
| **Frequency of daily eye exercises at school** | Once | 2047 (66.2%) | 4605 (68%) |  |  |
|  | Twice and more | 986 (31.9%) | 1937 (28.6%) | **0.87 (0.80-0.96, p=.004)** | **0.83 (0.75-0.92, p<.001)** |
|  | No | 60 (1.9%) | 230 (3.4%) | 1.70 (1.28-2.27, p<.001) | 1.01 (0.74-1.37, p=.969) |
| **Recess activity space** | Inside the teaching building | 602 (19.5%) | 1823 (26.9%) |  |  |
|  | Outdoor | 2491 (80.5%) | 4949 (73.1%) | **0.66 (0.59-0.73, p<.001)** | **0.82 (0.73-0.92, p=.001)** |
| **Average daily time spent doing homework or reading and writing after school** | <1 h | 1202 (38.9%) | 2144 (31.7%) |  |  |
|  | 1-2 h | 1130 (36.5%) | 2628 (38.8%) | 1.30 (1.18-1.44, p<.001) | 1.03 (0.92-1.14, p=.651) |
|  | ≥2 h | 493 (15.9%) | 1641 (24.2%) | **1.87 (1.65-2.11, p<.001)** | **1.17 (1.02-1.34, p=.022)** |
|  | No | 268 (8.7%) | 359 (5.3%) | 0.75 (0.63-0.89, p=.001) | 0.85 (0.71-1.03, p=.093) |
| **Average duration of extracurricular tuition per week** | No | 1373 (44.4%) | 3021 (44.6%) |  |  |
|  | <3 h | 1315 (42.5%) | 2755 (40.7%) | 0.95 (0.87-1.04, p=.294) |  |
|  | ≥3 h | 405 (13.1%) | 996 (14.7%) | 1.12 (0.98-1.28, p=.098) |  |
| **Read and write with your chest more than one punch away from the table** | No | 1342 (43.4%) | 3100 (45.8%) |  |  |
|  | Yes | 1751 (56.6%) | 3672 (54.2%) | 0.91 (0.83-0.99, p=.027) | 1.08 (0.86-1.34, p=.081) |
| **Read and write with eyes more than one foot away from the book** | No | 1193 (38.6%) | 3030 (44.7%) |  |  |
|  | Yes | 1900 (61.4%) | 3742 (55.3%) | **0.78 (0.71-0.85, p<.001)** | **0.75 (0.65-0.86, p<.001)** |
| **Read and write with fingers one inch from the tip of the pen** | No | 1046 (33.8%) | 2488 (36.7%) |  |  |
|  | Yes | 2047 (66.2%) | 4284 (63.3%) | 0.88 (0.80-0.96, p=.005) | 1.05 (0.93-1.19, p=.425) |
| **Average daily computer hours** | No | 1717 (55.5%) | 3714 (54.8%) |  |  |
|  | <1 h | 909 (29.4%) | 1952 (28.8%) | 0.99 (0.90-1.09, p=.884) |  |
|  | ≥1h | 467 (15.1%) | 1106 (16.3%) | 1.09 (0.97-1.24, p=.146) |  |
| **Hours of mobile electronic devices use** | No | 1301 (42.1%) | 2680 (39.6%) |  |  |
|  | <0.5 h | 461 (14.9%) | 1020 (15.1%) | 1.07 (0.94-1.22, p=.275) | 1.11 (0.97-1.28, p=.128) |
|  | ≥0.5 h | 1331 (43%) | 3072 (45.4%) | 1.12 (1.02-1.23, p=.016) | 1.00 (0.91-1.11, p=.969) |
| **Reading a book or electronic screen in direct sunlight** | No / Occasionally | 2945 (95.2%) | 6367 (94%) |  |  |
|  | Often | 111 (3.6%) | 283 (4.2%) | 1.18 (0.94-1.48, p=.149) | 0.89 (0.69-1.15, p=.388) |
|  | Always | 37 (1.2%) | 122 (1.8%) | 1.53 (1.05-2.21, p=.025) | 1.33 (0.86-2.04, p=.197) |
| **Reading the electronic screen with the lights off after dark** | No / Occasionally | 2894 (93.6%) | 6194 (91.5%) |  |  |
|  | Often | 136 (4.4%) | 392 (5.8%) | 1.35 (1.10-1.64, p=.004) | 1.02 (0.80-1.30, p=.846) |
|  | Always | 63 (2%) | 186 (2.7%) | 1.38 (1.03-1.84, p=.029) | 1.17 (0.83-1.65, p=.365) |
| **Reading a book or electronic screen while lying down or lying on the back** | No / Occasionally | 2649 (85.6%) | 5581 (82.4%) |  |  |
|  | Often | 378 (12.2%) | 1000 (14.8%) | 1.26 (1.11-1.43, p<.001) | 1.00 (0.86-1.16, p=.983) |
|  | Always | 66 (2.1%) | 191 (2.8%) | 1.37 (1.03-1.82, p=.028) | 1.05 (0.74-1.49, p=.801) |
| **Reading a book or electronic screen while walking or riding in a car** | No / Occasionally | 2952 (95.4%) | 6294 (92.9%) |  |  |
|  | Often | 113 (3.7%) | 377 (5.6%) | 1.56 (1.26-1.94, p<.001) | 0.92 (0.71-1.19, p=.526) |
|  | Always | 28 (0.9%) | 101 (1.5%) | 1.69 (1.11-2.58, p=.014) | 0.90 (0.52-1.55, p=.712) |
| **When using the computer, eyes from the screen more than 66cm** | No / Occasionally | 896 (29%) | 2230 (32.9%) |  |  |
|  | Often / Always | 2197 (71%) | 4542 (67.1%) | 0.83 (0.76-0.91, p<.001) | 0.94 (0.85-1.05, p=.299) |
| **When using eyes at close range, how often to rest your eyes** | < 15 min | 810 (26.2%) | 1595 (23.6%) |  |  |
|  | ≤ 15 < 30 min | 967 (31.3%) | 2150 (31.7%) | 1.13 (1.01-1.26, p=.036) | 1.05 (0.93-1.18, p=.475) |
|  | ≤ 30 <60 min | 474 (15.3%) | 1324 (19.6%) | 1.42 (1.24-1.62, p<.001) | 1.10 (0.98-1.36, p=.061) |
|  | ≥ 60 min | 842 (27.2%) | 1703 (25.1%) | 1.03 (0.91-1.16, p=.657) | 0.94 (0.83-1.07, p=.376) |
| **Daytime outdoor activity hours** | < 1 h | 675 (21.8%) | 1735 (25.6%) |  |  |
|  | 1-2 h | 1260 (40.7%) | 2788 (41.2%) | 0.86 (0.77-0.96, p=.008) | 0.93 (0.82-1.05, p=.236) |
|  | ≥ 2 h | 1158 (37.4%) | 2249 (33.2%) | **0.76 (0.67-0.85, p<.001)** | **0.86 (0.76-0.97, p=.018)** |
| **Average daily sleep duration** | < 8 h | 900 (29.1%) | 3051 (45.1%) |  |  |
|  | ≥ 8 h | 2193 (70.9%) | 3721 (54.9%) | 0.50 (0.46-0.55, p<.001) | 0.95 (0.85-1.06, p=.347) |

**Supplementary Table 3 Analysis of** **associated factors for myopia among primary and secondary school students in Shenyang in 2021** **(subgroup analysis-Female)**

| **Variables** | **Levels** | **Non-myopia**  (N=2353) | **Myopia**  (N=7664) | OR **(univariate)** | OR **(****multivariate)** |
| --- | --- | --- | --- | --- | --- |
| **Educational stage** | Primary 4-6th grade | 1253 (53.3%) | 2237 (29.2%) |  |  |
|  | Middle school | 606 (25.8%) | 2665 (34.8%) | **2.46 (2.20-2.76, p<.001)** | **2.53 (2.22-2.87, p<.001)** |
|  | General high school | 242 (10.3%) | 2139 (27.9%) | **4.95 (4.26-5.75, p<.001)** | **4.65 (3.88-5.58, p<.001)** |
|  | Vocational high school | 252 (10.7%) | 623 (8.1%) | **1.38 (1.18-1.63, p<.001)** | **1.37 (1.12-1.68, p=.002)** |
| **Region** | Urban | 1787 (75.9%) | 5714 (74.6%) |  |  |
|  | Rural | 566 (24.1%) | 1950 (25.4%) | 1.08 (0.97-1.20, p=.174) |  |
| **Ethnicity** | Han | 1790 (76.1%) | 5932 (77.4%) |  |  |
|  | Others | 563 (23.9%) | 1732 (22.6%) | 0.93 (0.83-1.03, p=.180) |  |
| **Family genetic history** | Neither parent is myopic | 1649 (70.1%) | 4161 (54.3%) |  |  |
|  | Father is myopic | 219 (9.3%) | 1083 (14.1%) | **1.96 (1.68-2.29, p<.001)** | **2.13 (1.81-2.51, p<.001)** |
|  | Mother is myopic | 334 (14.2%) | 1553 (20.3%) | **1.84 (1.62-2.10, p<.001)** | **2.06 (1.80-2.37, p<.001)** |
|  | Both parents are myopic | 151 (6.4%) | 867 (11.3%) | **2.28 (1.90-2.73, p<.001)** | **2.74 (2.26-3.32, p<.001)** |
| **Frequency of changing seat** | Weekly | 1331 (56.6%) | 4025 (52.5%) |  |  |
|  | Biweekly | 352 (15%) | 1172 (15.3%) | 1.10 (0.96-1.26, p=.160) | 1.04 (0.90-1.20, p=.594) |
|  | Once a month/no change | 670 (28.5%) | 2467 (32.2%) | 1.22 (1.10-1.35, p<.001) | 1.00 (0.89-1.13, p=.987) |
| **Adjust the height of the desk and chair according to the height** | Never or non-adjustable | 846 (36%) | 3197 (41.7%) |  |  |
|  | Once a year | 323 (13.7%) | 990 (12.9%) | 0.81 (0.70-0.94, p=.005) | 0.97 (0.83-1.13, p=.693) |
|  | Once a term | 675 (28.7%) | 1962 (25.6%) | 0.77 (0.69-0.86, p<.001) | 1.03 (0.90-1.17, p=.684) |
|  | Once every 2 to 3 months | 509 (21.6%) | 1515 (19.8%) | 0.79 (0.69-0.89, p<.001) | 0.99 (0.86-1.14, p=.909) |
| **Frequency of daily eye exercises at school** | Once | 1471 (62.5%) | 5010 (65.4%) |  |  |
|  | Twice and more | 784 (33.3%) | 2169 (28.3%) | **0.81 (0.73-0.90, p<.001)** | **0.73 (0.66-0.82, p<.001)** |
|  | No | 98 (4.2%) | 485 (6.3%) | 1.45 (1.16-1.82, p=.001) | 1.21 (0.93-1.58, p=.160) |
| **Recess activity space** | Inside the teaching building | 634 (26.9%) | 2651 (34.6%) |  |  |
|  | Outdoor | 1719 (73.1%) | 5013 (65.4%) | **0.70 (0.63-0.77, p<.001)** | **0.89 (0.79-1.00, p=.050)** |
| **Average daily time spent doing homework or reading and writing after school** | <1 h | 864 (36.7%) | 2292 (29.9%) |  |  |
|  | 1-2 h | 886 (37.7%) | 3035 (39.6%) | 1.29 (1.16-1.44, p<.001) | 0.98 (0.87-1.10, p=.757) |
|  | ≥2 h | 410 (17.4%) | 1991 (26%) | 1.83 (1.60-2.09, p<.001) | 1.08 (0.93-1.25, p=.331) |
|  | No | 193 (8.2%) | 346 (4.5%) | **0.68 (0.56-0.82, p<.001)** | **0.79 (0.64-0.97, p=.022)** |
| **Average duration of extracurricular tuition per week** | No | 1051 (44.7%) | 3436 (44.8%) |  |  |
|  | <3 h | 1002 (42.6%) | 3128 (40.8%) | 0.95 (0.86-1.05, p=.361) |  |
|  | ≥3 h | 300 (12.7%) | 1100 (14.4%) | 1.12 (0.97-1.30, p=.121) |  |
| **Read and write with your chest more than one punch away from the table** | No | 1043 (44.3%) | 3835 (50%) |  |  |
|  | Yes | 1310 (55.7%) | 3829 (50%) | 0.79 (0.72-0.87, p<.001) | 1.01 (0.88-1.16, p=.854) |
| **Read and write with eyes more than one foot away from the book** | No | 965 (41%) | 3751 (48.9%) |  |  |
|  | Yes | 1388 (59%) | 3913 (51.1%) | **0.73 (0.66-0.80, p<.001)** | **0.75 (0.65-0.87, p<.001)** |
| **Read and write with fingers one inch from the tip of the pen** | No | 832 (35.4%) | 2937 (38.3%) |  |  |
|  | Yes | 1521 (64.6%) | 4727 (61.7%) | 0.88 (0.80-0.97, p=.009) | 1.13 (0.99-1.29, p=.060) |
| **Average daily computer hours** | No | 1387 (58.9%) | 4491 (58.6%) |  |  |
|  | <1 h | 674 (28.6%) | 2115 (27.6%) | 0.97 (0.87-1.08, p=.560) |  |
|  | ≥1h | 292 (12.4%) | 1058 (13.8%) | 1.12 (0.97-1.29, p=.123) |  |
| **Hours of mobile electronic devices use** | No | 960 (40.8%) | 2862 (37.3%) |  |  |
|  | <0.5 h | 375 (15.9%) | 1154 (15.1%) | 1.03 (0.90-1.18, p=.651) | 0.94 (0.76-1.18, p=.611) |
|  | ≥0.5 h | 1018 (43.3%) | 3648 (47.6%) | 1.20 (1.09-1.33, p<.001) | 1.00 (0.82-1.21, p=.966) |
| **Reading a book or electronic screen in direct sunlight** | No / Occasionally | 2247 (95.5%) | 7307 (95.3%) |  |  |
|  | Often | 81 (3.4%) | 279 (3.6%) | 1.06 (0.82-1.36, p=.654) |  |
|  | Always | 25 (1.1%) | 78 (1%) | 0.96 (0.61-1.51, p=.858) |  |
| **Reading the electronic screen with the lights off after dark** | No / Occasionally | 2183 (92.8%) | 6901 (90%) |  |  |
|  | Often | 111 (4.7%) | 533 (7%) | 1.52 (1.23-1.87, p<.001) | 1.15 (0.90-1.47, p=.258) |
|  | Always | 59 (2.5%) | 230 (3%) | 1.23 (0.92-1.65, p=.157) | 1.05 (0.74-1.47, p=.791) |
| **Reading a book or electronic screen while lying down or lying on the back** | No / Occasionally | 2045 (86.9%) | 6285 (82%) |  |  |
|  | Often | 248 (10.5%) | 1157 (15.1%) | 1.52 (1.31-1.76, p<.001) | 1.18 (0.98-1.43, p=.057) |
|  | Always | 60 (2.5%) | 222 (2.9%) | 1.20 (0.90-1.61, p=.209) | 0.71 (0.49-1.03, p=.073) |
| **Reading a book or electronic screen while walking or riding in a car** | No / Occasionally | 2225 (94.6%) | 7078 (92.4%) |  |  |
|  | Often | 106 (4.5%) | 465 (6.1%) | 1.38 (1.11-1.71, p=.004) | 1.04 (0.74-1.46, p=.819) |
|  | Always | 22 (0.9%) | 121 (1.6%) | 1.73 (1.10-2.73, p=.019) | 1.60 (0.92-2.79, p=.096) |
| **When using the computer, eyes from the screen more than 66cm** | No / Occasionally | 724 (30.8%) | 2571 (33.5%) |  |  |
|  | Often / Always | 1629 (69.2%) | 5093 (66.5%) | 0.88 (0.80-0.97, p=.012) | 1.00 (0.89-1.12, p=.974) |
| **When using eyes at close range, how often to rest your eyes** | < 15 min | 595 (25.3%) | 1760 (23%) |  |  |
|  | ≤ 15 < 30 min | 751 (31.9%) | 2499 (32.6%) | 1.12 (0.99-1.27, p=.062) | 1.00 (0.88-1.15, p=.943) |
|  | ≤ 30 <60 min | 361 (15.3%) | 1345 (17.5%) | 1.26 (1.09-1.46, p=.002) | 0.93 (0.79-1.09, p=.349) |
|  | ≥ 60 min | 646 (27.5%) | 2060 (26.9%) | 1.08 (0.95-1.23, p=.251) | 0.94 (0.81-1.07, p=.341) |
| **Daytime outdoor activity hours** | < 1 h | 620 (26.3%) | 2353 (30.7%) |  |  |
|  | 1-2 h | 1033 (43.9%) | 3154 (41.2%) | 0.80 (0.72-0.90, p<.001) | 0.92 (0.81-1.04, p=.164) |
|  | ≥ 2 h | 700 (29.7%) | 2157 (28.1%) | 0.81 (0.72-0.92, p<.001) | 0.99 (0.87-1.13, p=.887) |
| **Average daily sleep duration** | < 8 h | 696 (29.6%) | 3688 (48.1%) |  |  |
|  | ≥ 8 h | 1657 (70.4%) | 3976 (51.9%) | **0.45 (0.41-0.50, p<.001)** | **0.87 (0.77-0.99, p=.033)** |

**Supplementary Table 4 Analysis of associated factors for myopia among primary and secondary school students in Shenyang in 2021 (subgroup analysis-Primary school)**

| **Variables** | **Levels** | **Non-myopia**  (N=2946) | **Myopia**  (N=4322) | OR (univariate) | OR (multivariate) |
| --- | --- | --- | --- | --- | --- |
| **Sex** | Male | 1693 (57.5%) | 2085 (48.2%) |  |  |
|  | Female | 1253 (42.5%) | 2237 (51.8%) | **1.45 (1.32-1.59, p<.001)** | **1.46 (1.32-1.61, p<.001)** |
| **Region** | Urban | 2140 (72.6%) | 2915 (67.4%) |  |  |
|  | Rural | 806 (27.4%) | 1407 (32.6%) | **1.28 (1.16-1.42, p<.001)** | **1.53 (1.37-1.71, p<.001)** |
| **Ethnicity** | Han | 2253 (76.5%) | 3350 (77.5%) |  |  |
|  | Others | 693 (23.5%) | 972 (22.5%) | 0.94 (0.84-1.05, p=.303) |  |
| **Family genetic history** | Neither parent is myopic | 1869 (63.4%) | 1868 (43.2%) |  |  |
|  | Father is myopic | 308 (10.5%) | 656 (15.2%) | **2.13 (1.83-2.48, p<.001)** | **2.23 (1.92-2.60, p<.001)** |
|  | Mother is myopic | 525 (17.8%) | 1073 (24.8%) | **2.04 (1.81-2.31, p<.001)** | **2.11 (1.86-2.39, p<.001)** |
|  | Both parents are myopic | 244 (8.3%) | 725 (16.8%) | **2.97 (2.54-3.48, p<.001)** | **3.15 (2.68-3.70, p<.001)** |
| **Frequency of changing seat** | Weekly | 1956 (66.4%) | 2810 (65%) |  |  |
|  | Biweekly | 406 (13.8%) | 662 (15.3%) | 1.13 (0.99-1.30, p=.069) |  |
|  | Once a month/no change | 584 (19.8%) | 850 (19.7%) | 1.01 (0.90-1.14, p=.831) |  |
| **Adjust the height of the desk and chair according to the height** | Never or non-adjustable | 835 (28.3%) | 1273 (29.5%) |  |  |
|  | Once a year | 428 (14.5%) | 647 (15%) | 0.99 (0.85-1.15, p=.912) |  |
|  | Once a term | 1000 (33.9%) | 1472 (34.1%) | 0.97 (0.86-1.09, p=.562) |  |
|  | Once every 2 to 3 months | 683 (23.2%) | 930 (21.5%) | 0.89 (0.78-1.02, p=.093) |  |
| **Frequency of daily eye exercises at school** | Once | 1996 (67.8%) | 3090 (71.5%) |  |  |
|  | Twice and more | 943 (32%) | 1210 (28%) | **0.83 (0.75-0.92, p<.001)** | **0.74 (0.67-0.83, p<.001)** |
|  | No | 7 (0.2%) | 22 (0.5%) | 2.03 (0.87-4.76, p=.103) | 1.97 (0.82-4.75, p=.129) |
| **Recess activity space** | Inside the teaching building | 445 (15.1%) | 748 (17.3%) |  |  |
|  | Outdoor | 2501 (84.9%) | 3574 (82.7%) | 0.85 (0.75-0.97, p=.013) | 0.94 (0.82-1.08, p=.379) |
| **Average daily time spent doing homework or reading and writing after school** | <1 h | 1396 (47.4%) | 2026 (46.9%) |  |  |
|  | 1-2 h | 914 (31%) | 1425 (33%) | 1.07 (0.96-1.20, p=.191) | 1.02 (0.91-1.14, p=.712) |
|  | ≥2 h | 270 (9.2%) | 472 (10.9%) | 1.20 (1.02-1.42, p=.026) | 1.13 (0.95-1.34, p=.172) |
|  | No | 366 (12.4%) | 399 (9.2%) | **0.75 (0.64-0.88, p<.001)** | **0.77 (0.65-0.90, p=.002)** |
| **Average duration of extracurricular tuition per week** | No | 1154 (39.2%) | 1548 (35.8%) |  |  |
|  | <3 h | 1403 (47.6%) | 2155 (49.9%) | 1.15 (1.03-1.27, p=.009) | 1.09 (0.98-1.22, p=.117) |
|  | ≥3 h | 389 (13.2%) | 619 (14.3%) | 1.19 (1.02-1.38, p=.024) | 1.01 (0.86-1.18, p=.929) |
| **Read and write with your chest more than one punch away from the table** | No | 1265 (42.9%) | 2030 (47%) |  |  |
|  | Yes | 1681 (57.1%) | 2292 (53%) | 0.85 (0.77-0.93, p<.001) | 1.09 (0.95-1.25, p=.219) |
| **Read and write with eyes more than one foot away from the book** | No | 1135 (38.5%) | 1979 (45.8%) |  |  |
|  | Yes | 1811 (61.5%) | 2343 (54.2%) | **0.74 (0.67-0.82, p<.001)** | **0.73 (0.63-0.84, p<.001)** |
| **Read and write with fingers one inch from the tip of the pen** | No | 967 (32.8%) | 1551 (35.9%) |  |  |
|  | Yes | 1979 (67.2%) | 2771 (64.1%) | 0.87 (0.79-0.96, p=.007) | 1.09 (0.96-1.25, p=.180) |
| **Average daily computer hours** | No | 1824 (61.9%) | 2713 (62.8%) |  |  |
|  | <1 h | 860 (29.2%) | 1189 (27.5%) | 0.93 (0.84-1.03, p=.176) |  |
|  | ≥1h | 262 (8.9%) | 420 (9.7%) | 1.08 (0.91-1.27, p=.375) |  |
| **Hours of mobile electronic devices use** | No | 1269 (43.1%) | 1809 (41.9%) |  |  |
|  | <0.5 h | 568 (19.3%) | 917 (21.2%) | 1.13 (1.00-1.29, p=.055) |  |
|  | ≥0.5 h | 1109 (37.6%) | 1596 (36.9%) | 1.01 (0.91-1.12, p=.859) |  |
| **Reading a book or electronic screen in direct sunlight** | No / Occasionally | 2870 (97.4%) | 4201 (97.2%) |  |  |
|  | Often | 54 (1.8%) | 87 (2%) | 1.10 (0.78-1.55, p=.584) |  |
|  | Always | 22 (0.7%) | 34 (0.8%) | 1.06 (0.62-1.81, p=.843) |  |
| **Reading the electronic screen with the lights off after dark** | No / Occasionally | 2836 (96.3%) | 4156 (96.2%) |  |  |
|  | Often | 62 (2.1%) | 96 (2.2%) | 1.06 (0.77-1.46, p=.738) |  |
|  | Always | 48 (1.6%) | 70 (1.6%) | 1.00 (0.69-1.44, p=.980) |  |
| **Reading a book or electronic screen while lying down or lying on the back** | No / Occasionally | 2671 (90.7%) | 3835 (88.7%) |  |  |
|  | Often | 242 (8.2%) | 438 (10.1%) | 1.26 (1.07-1.49, p=.006) | 1.14 (0.96-1.36, p=.128) |
|  | Always | 33 (1.1%) | 49 (1.1%) | 1.03 (0.66-1.61, p=.882) | 1.05 (0.66-1.66, p=.842) |
| **Reading a book or electronic screen while walking or riding in a car** | No / Occasionally | 2907 (98.7%) | 4250 (98.3%) |  |  |
|  | Often | 31 (1.1%) | 58 (1.3%) | 1.28 (0.83-1.98, p=.270) |  |
|  | Always | 8 (0.3%) | 14 (0.3%) | 1.20 (0.50-2.86, p=.685) |  |
| **When using the computer, eyes from the screen more than 66cm** | No / Occasionally | 787 (26.7%) | 1275 (29.5%) |  |  |
|  | Often / Always | 2159 (73.3%) | 3047 (70.5%) | 0.87 (0.78-0.97, p=.010) | 0.94 (0.84-1.05, p=.287) |
| **When using eyes at close range, how often to rest your eyes** | < 15 min | 804 (27.3%) | 1160 (26.8%) |  |  |
|  | ≤ 15 < 30 min | 1026 (34.8%) | 1566 (36.2%) | 1.06 (0.94-1.19, p=.356) |  |
|  | ≤ 30 <60 min | 397 (13.5%) | 649 (15%) | 1.13 (0.97-1.32, p=.112) |  |
|  | ≥ 60 min | 719 (24.4%) | 947 (21.9%) | 0.91 (0.80-1.04, p=.177) |  |
| **Daytime outdoor activity hours** | < 1 h | 659 (22.4%) | 1042 (24.1%) |  |  |
|  | 1-2 h | 1268 (43%) | 1858 (43%) | 0.93 (0.82-1.05, p=.217) |  |
|  | ≥ 2 h | 1019 (34.6%) | 1422 (32.9%) | 0.88 (0.78-1.00, p=.053) |  |
| **Average daily sleep duration** | < 8 h | 352 (11.9%) | 517 (12%) |  |  |
|  | ≥ 8 h | 2594 (88.1%) | 3805 (88%) | 1.00 (0.86-1.15, p=.986) |  |
|  | | | | | |

**Supplementary Table 5 Analysis of associated factors for myopia among primary and secondary school students in Shenyang in 2021 (subgroup analysis-Middle school)**

| **Variables** | **Levels** | **Non-myopia**  (N=1578) | **Myopia**  (N=5240) | OR (univariate) | OR (multivariate) |
| --- | --- | --- | --- | --- | --- |
| **Sex** | Male | 972 (61.6%) | 2575 (49.1%) |  |  |
|  | Female | 606 (38.4%) | 2665 (50.9%) | **1.66 (1.48-1.86, p<.001)** | **1.60 (1.42-1.80, p<.001)** |
| **Region** | Urban | 1129 (71.5%) | 3590 (68.5%) |  |  |
|  | Rural | 449 (28.5%) | 1650 (31.5%) | **1.16 (1.02-1.31, p=.022)** | **1.30 (1.14-1.48, p<.001)** |
| **Ethnicity** | Han | 1235 (78.3%) | 4004 (76.4%) |  |  |
|  | Others | 343 (21.7%) | 1236 (23.6%) | 1.11 (0.97-1.27, p=.126) |  |
| **Family genetic history** | Neither parent is myopic | 1195 (75.7%) | 3049 (58.2%) |  |  |
|  | Father is myopic | 123 (7.8%) | 736 (14%) | **2.35 (1.92-2.87, p<.001)** | **2.33 (1.89-2.86, p<.001)** |
|  | Mother is myopic | 201 (12.7%) | 1011 (19.3%) | **1.97 (1.67-2.33, p<.001)** | **1.96 (1.66-2.32, p<.001)** |
|  | Both parents are myopic | 59 (3.7%) | 444 (8.5%) | **2.95 (2.23-3.90, p<.001)** | **2.85 (2.15-3.79, p<.001)** |
| **Frequency of changing seat** | Weekly | 847 (53.7%) | 2966 (56.6%) |  |  |
|  | Biweekly | 235 (14.9%) | 705 (13.5%) | 0.86 (0.73-1.01, p=.068) |  |
|  | Once a month/no change | 496 (31.4%) | 1569 (29.9%) | 0.90 (0.80-1.03, p=.116) |  |
| **Adjust the height of the desk and chair according to the height** | Never or non-adjustable | 572 (36.2%) | 1976 (37.7%) |  |  |
|  | Once a year | 190 (12%) | 600 (11.5%) | 0.91 (0.76-1.10, p=.349) |  |
|  | Once a term | 393 (24.9%) | 1320 (25.2%) | 0.97 (0.84-1.13, p=.706) |  |
|  | Once every 2 to 3 months | 423 (26.8%) | 1344 (25.6%) | 0.92 (0.80-1.06, p=.253) |  |
| **Frequency of daily eye exercises at school** | Once | 1029 (65.2%) | 3628 (69.2%) |  |  |
|  | Twice and more | 494 (31.3%) | 1475 (28.1%) | **0.85 (0.75-0.96, p=.008)** | **0.71 (0.62-0.81, p<.001)** |
|  | No | 55 (3.5%) | 137 (2.6%) | 0.71 (0.51-0.97, p=.034) | 0.87 (0.73-1.03, p=.112) |
| **Recess activity space** | Inside the teaching building | 435 (27.6%) | 1700 (32.4%) |  |  |
|  | Outdoor | 1143 (72.4%) | 3540 (67.6%) | 0.79 (0.70-0.90, p<.001) | 0.88 (0.77-1.01, p=.060) |
| **Average daily time spent doing homework or reading and writing after school** | <1 h | 411 (26%) | 1307 (24.9%) |  |  |
|  | 1-2 h | 736 (46.6%) | 2278 (43.5%) | 0.97 (0.85-1.12, p=.702) | 0.94 (0.81-1.08, p=.368) |
|  | ≥2 h | 374 (23.7%) | 1507 (28.8%) | 1.27 (1.08-1.48, p=.003) | 1.15 (0.97-1.37, p=.099) |
|  | No | 57 (3.6%) | 148 (2.8%) | 0.82 (0.59-1.13, p=.222) | 0.85 (0.61-1.19, p=.351) |
| **Average duration of extracurricular tuition per week** | No | 840 (53.2%) | 2791 (53.3%) |  |  |
|  | <3 h | 553 (35%) | 1814 (34.6%) | 0.99 (0.87-1.12, p=.838) |  |
|  | ≥3 h | 185 (11.7%) | 635 (12.1%) | 1.03 (0.86-1.24, p=.725) |  |
| **Read and write with your chest more than one punch away from the table** | No | 663 (42%) | 2399 (45.8%) |  |  |
|  | Yes | 915 (58%) | 2841 (54.2%) | 0.86 (0.77-0.96, p=.008) | 1.09 (0.93-1.29, p=.287) |
| **Read and write with eyes more than one foot away from the book** | No | 601 (38.1%) | 2396 (45.7%) |  |  |
|  | Yes | 977 (61.9%) | 2844 (54.3%) | **0.73 (0.65-0.82, p<.001)** | **0.73 (0.61-0.87, p<.001)** |
| **Read and write with fingers one inch from the tip of the pen** | No | 536 (34%) | 1932 (36.9%) |  |  |
|  | Yes | 1042 (66%) | 3308 (63.1%) | 0.88 (0.78-0.99, p=.035) | 1.07 (0.91-1.26, p=.387) |
| **Average daily computer hours** | No | 838 (53.1%) | 2953 (56.4%) |  |  |
|  | <1 h | 457 (29%) | 1538 (29.4%) | 0.96 (0.84-1.09, p=.486) | 0.91 (0.80-1.05, p=.201) |
|  | ≥1h | 283 (17.9%) | 749 (14.3%) | 0.75 (0.64-0.88, p<.001) | 0.85 (0.70-1.02, p=.086) |
| **Hours of mobile electronic devices use** | No | 628 (39.8%) | 1944 (37.1%) |  |  |
|  | <0.5 h | 188 (11.9%) | 727 (13.9%) | 1.25 (1.04-1.50, p=.018) | 1.16 (0.96-1.40, p=.133) |
|  | ≥0.5 h | 762 (48.3%) | 2569 (49%) | 1.09 (0.97-1.23, p=.167) | 1.13 (1.00-1.29, p=.059) |
| **Reading a book or electronic screen in direct sunlight** | No / Occasionally | 1458 (92.4%) | 4930 (94.1%) |  |  |
|  | Often | 93 (5.9%) | 226 (4.3%) | 0.72 (0.56-0.92, p=.009) | 0.78 (0.60-1.01, p=.054) |
|  | Always | 27 (1.7%) | 84 (1.6%) | 0.92 (0.59-1.43, p=.709) | 1.05 (0.65-1.70, p=.847) |
| **Reading the electronic screen with the lights off after dark** | No / Occasionally | 1431 (90.7%) | 4815 (91.9%) |  |  |
|  | Often | 103 (6.5%) | 278 (5.3%) | 0.80 (0.64-1.01, p=.064) |  |
|  | Always | 44 (2.8%) | 147 (2.8%) | 0.99 (0.71-1.40, p=.967) |  |
| **Reading a book or electronic screen while lying down or lying on the back** | No / Occasionally | 1299 (82.3%) | 4360 (83.2%) |  |  |
|  | Often | 227 (14.4%) | 736 (14%) | 0.97 (0.82-1.13, p=.674) |  |
|  | Always | 52 (3.3%) | 144 (2.7%) | 0.83 (0.60-1.14, p=.243) |  |
| **Reading a book or electronic screen while walking or riding in a car** | No / Occasionally | 1482 (93.9%) | 4988 (95.2%) |  |  |
|  | Often | 77 (4.9%) | 195 (3.7%) | 0.75 (0.57-0.99, p=.039) | 0.81 (0.60-1.08, p=.151) |
|  | Always | 19 (1.2%) | 57 (1.1%) | 0.89 (0.53-1.50, p=.666) | 0.98 (0.55-1.76, p=.954) |
| **When using the computer, eyes from the screen more than 66cm** | No / Occasionally | 479 (30.4%) | 1731 (33%) |  |  |
|  | Often / Always | 1099 (69.6%) | 3509 (67%) | 0.88 (0.78-1.00, p=.046) | 0.93 (0.81-1.07, p=.300) |
| **When using eyes at close range, how often to rest your eyes** | < 15 min | 391 (24.8%) | 1237 (23.6%) |  |  |
|  | ≤ 15 < 30 min | 445 (28.2%) | 1663 (31.7%) | 1.18 (1.01-1.38, p=.035) | 1.10 (0.94-1.30, p=.227) |
|  | ≤ 30 <60 min | 263 (16.7%) | 947 (18.1%) | 1.14 (0.95-1.36, p=.154) | 1.10 (0.91-1.32, p=.330) |
|  | ≥ 60 min | 479 (30.4%) | 1393 (26.6%) | 0.92 (0.79-1.07, p=.284) | 0.90 (0.76-1.05, p=.188) |
| **Daytime outdoor activity hours** | < 1 h | 397 (25.2%) | 1613 (30.8%) |  |  |
|  | 1-2 h | 656 (41.6%) | 2066 (39.4%) | **0.78 (0.67-0.89, p<.001)** | **0.81 (0.70-0.94, p=.005)** |
|  | ≥ 2 h | 525 (33.3%) | 1561 (29.8%) | 0.73 (0.63-0.85, p<.001) | 0.86 (0.73-1.01, p=.067) |
| **Average daily sleep duration** | < 8 h | 644 (40.8%) | 2373 (45.3%) |  |  |
|  | ≥ 8 h | 934 (59.2%) | 2867 (54.7%) | 0.83 (0.74-0.93, p=.002) | 0.91 (0.80-1.03, p=.140) |
|  | | | | | |

**Supplementary Table 6 Analysis of associated factors for myopia among primary and secondary school students in Shenyang in 2021 (subgroup analysis-General high school)**

| **Variables** | **Levels** | **Non-myopia**  (N=586) | **Myopia** (N=4102) | OR (univariate) | OR (multivariate) |
| --- | --- | --- | --- | --- | --- |
| **Sex** | Male | 344 (58.7%) | 1963 (47.9%) |  |  |
|  | Female | 242 (41.3%) | 2139 (52.1%) | **1.55 (1.30-1.85, p<.001)** | **1.48 (1.23-1.77, p<.001)** |
| **Region** | Urban | 474 (80.9%) | 3396 (82.8%) |  |  |
|  | Rural | 112 (19.1%) | 706 (17.2%) | 0.88 (0.71-1.10, p=.257) |  |
| **Ethnicity** | Han | 442 (75.4%) | 3191 (77.8%) |  |  |
|  | Others | 144 (24.6%) | 911 (22.2%) | 0.88 (0.72-1.07, p=.200) |  |
| **Family genetic history** | Neither parent is myopic | 434 (74.1%) | 2323 (56.6%) |  |  |
|  | Father is myopic | 57 (9.7%) | 588 (14.3%) | **1.93 (1.44-2.58, p<.001)** | **1.89 (1.41-2.54, p<.001)** |
|  | Mother is myopic | 73 (12.5%) | 780 (19%) | **2.00 (1.54-2.59, p<.001)** | **1.96 (1.51-2.56, p<.001)** |
|  | Both parents are myopic | 22 (3.8%) | 411 (10%) | **3.49 (2.25-5.42, p<.001)** | **3.32 (2.13-5.17, p<.001)** |
| **Frequency of changing seat** | Weekly | 279 (47.6%) | 1976 (48.2%) |  |  |
|  | Biweekly | 81 (13.8%) | 593 (14.5%) | 1.03 (0.79-1.35, p=.806) |  |
|  | Once a month/no change | 226 (38.6%) | 1533 (37.4%) | 0.96 (0.79-1.16, p=.652) |  |
| **Adjust the height of the desk and chair according to the height** | Never or non-adjustable | 261 (44.5%) | 2087 (50.9%) |  |  |
|  | Once a year | 78 (13.3%) | 459 (11.2%) | 0.74 (0.56-0.97, p=.027) | 0.77 (0.58-1.03, p=.075) |
|  | Once a term | 117 (20%) | 772 (18.8%) | 0.83 (0.65-1.04, p=.106) | 0.93 (0.73-1.19, p=.579) |
|  | Once every 2 to 3 months | 130 (22.2%) | 784 (19.1%) | 0.75 (0.60-0.95, p=.014) | 0.96 (0.75-1.22, p=.714) |
| **Frequency of daily eye exercises at school** | Once | 387 (66%) | 2706 (66%) |  |  |
|  | Twice and more | 169 (28.8%) | 1115 (27.2%) | 0.94 (0.78-1.15, p=.557) |  |
|  | No | 30 (5.1%) | 281 (6.9%) | 1.34 (0.91-1.98, p=.143) |  |
| **Recess activity space** | Inside the teaching building | 173 (29.5%) | 1515 (36.9%) |  |  |
|  | Outdoor | 413 (70.5%) | 2587 (63.1%) | **0.72 (0.59-0.86, p<.001)** | **0.82 (0.67-1.00, p=.050)** |
| **Average daily time spent doing homework or reading and writing after school** | <1 h | 143 (24.4%) | 853 (20.8%) |  |  |
|  | 1-2 h | 230 (39.2%) | 1660 (40.5%) | 1.21 (0.97-1.51, p=.096) |  |
|  | ≥2 h | 201 (34.3%) | 1487 (36.3%) | 1.24 (0.99-1.56, p=.067) |  |
|  | No | 12 (2%) | 102 (2.5%) | 1.42 (0.76-2.66, p=.266) |  |
| **Average duration of extracurricular tuition per week** | No | 238 (40.6%) | 1679 (40.9%) |  |  |
|  | <3 h | 241 (41.1%) | 1644 (40.1%) | 0.97 (0.80-1.17, p=.731) |  |
|  | ≥3 h | 107 (18.3%) | 779 (19%) | 1.03 (0.81-1.32, p=.800) |  |
| **Read and write with your chest more than one punch away from the table** | No | 265 (45.2%) | 2048 (49.9%) |  |  |
|  | Yes | 321 (54.8%) | 2054 (50.1%) | 0.83 (0.70-0.99, p=.033) | 1.14 (0.90-1.45, p=.273) |
| **Read and write with eyes more than one foot away from the book** | No | 230 (39.2%) | 1957 (47.7%) |  |  |
|  | Yes | 356 (60.8%) | 2145 (52.3%) | **0.71 (0.59-0.85, p<.001)** | **0.73 (0.57-0.93, p=.011)** |
| **Read and write with fingers one inch from the tip of the pen** | No | 206 (35.2%) | 1545 (37.7%) |  |  |
|  | Yes | 380 (64.8%) | 2557 (62.3%) | 0.90 (0.75-1.08, p=.240) |  |
| **Average daily computer hours** | No | 286 (48.8%) | 2209 (53.9%) |  |  |
|  | <1 h | 186 (31.7%) | 1162 (28.3%) | 0.81 (0.66-0.99, p=.035) | 0.84 (0.68-1.02, p=.080) |
|  | ≥1h | 114 (19.5%) | 731 (17.8%) | 0.83 (0.66-1.05, p=.117) | 0.88 (0.69-1.12, p=.298) |
| **Hours of mobile electronic devices use** | No | 237 (40.4%) | 1509 (36.8%) |  |  |
|  | <0.5 h | 69 (11.8%) | 513 (12.5%) | 1.17 (0.88-1.55, p=.288) |  |
|  | ≥0.5 h | 280 (47.8%) | 2080 (50.7%) | 1.17 (0.97-1.40, p=.103) |  |
| **Reading a book or electronic screen in direct sunlight** | No / Occasionally | 554 (94.5%) | 3848 (93.8%) |  |  |
|  | Often | 26 (4.4%) | 191 (4.7%) | 1.06 (0.70-1.61, p=.793) |  |
|  | Always | 6 (1%) | 63 (1.5%) | 1.51 (0.65-3.51, p=.336) |  |
| **Reading the electronic screen with the lights off after dark** | No / Occasionally | 520 (88.7%) | 3556 (86.7%) |  |  |
|  | Often | 47 (8%) | 413 (10.1%) | 1.28 (0.94-1.76, p=.119) |  |
|  | Always | 19 (3.2%) | 133 (3.2%) | 1.02 (0.63-1.67, p=.925) |  |
| **Reading a book or electronic screen while lying down or lying on the back** | No / Occasionally | 472 (80.5%) | 3165 (77.2%) |  |  |
|  | Often | 93 (15.9%) | 788 (19.2%) | 1.26 (1.00-1.60, p=.052) |  |
|  | Always | 21 (3.6%) | 149 (3.6%) | 1.06 (0.66-1.69, p=.813) |  |
| **Reading a book or electronic screen while walking or riding in a car** | No / Occasionally | 515 (87.9%) | 3520 (85.8%) |  |  |
|  | Often | 57 (9.7%) | 485 (11.8%) | 1.24 (0.93-1.66, p=.138) |  |
|  | Always | 14 (2.4%) | 97 (2.4%) | 1.01 (0.57-1.79, p=.963) |  |
| **When using the computer, eyes from the screen more than 66cm** | No / Occasionally | 194 (33.1%) | 1428 (34.8%) |  |  |
|  | Often / Always | 392 (66.9%) | 2674 (65.2%) | 0.93 (0.77-1.11, p=.417) |  |
| **When using eyes at close range, how often to rest your eyes** | < 15 min | 125 (21.3%) | 789 (19.2%) |  |  |
|  | ≤ 15 < 30 min | 180 (30.7%) | 1259 (30.7%) | 1.11 (0.87-1.42, p=.411) |  |
|  | ≤ 30 <60 min | 123 (21%) | 947 (23.1%) | 1.22 (0.93-1.59, p=.144) |  |
|  | ≥ 60 min | 158 (27%) | 1107 (27%) | 1.11 (0.86-1.43, p=.416) |  |
| **Daytime outdoor activity hours** | < 1 h | 151 (25.8%) | 1214 (29.6%) |  |  |
|  | 1-2 h | 233 (39.8%) | 1703 (41.5%) | 0.91 (0.73-1.13, p=.391) | 1.08 (0.86-1.36, p=.484) |
|  | ≥ 2 h | 202 (34.5%) | 1185 (28.9%) | 0.73 (0.58-0.91, p=.006) | 0.95 (0.74-1.20, p=.655) |
| **Average daily sleep duration** | < 8 h | 469 (80%) | 3429 (83.6%) |  |  |
|  | ≥ 8 h | 117 (20%) | 673 (16.4%) | **0.79 (0.63-0.98, p=.032)** | **0.89 (0.79-1.00, p=.050)** |
|  | | | | | |

**Supplementary Table 7 Analysis of associated factors for myopia among primary and secondary school students in Shenyang in 2021 (subgroup analysis-Vocational high school)**

| **Variables** | **Levels** | **Non-myopia (N=336)** | **Myopia**  **(N=772)** | **OR (univariate)** | **OR (multivariate)** |
| --- | --- | --- | --- | --- | --- |
| **Sex** | Male | 84 (25%) | 149 (19.3%) |  |  |
|  | Female | 252 (75%) | 623 (80.7%) | 1.39 (1.03-1.89, p=.033) | 1.22 (0.87-1.69, p=.245) |
| **Ethnicity** | Han | 279 (83%) | 616 (79.8%) |  |  |
|  | Others | 57 (17%) | 156 (20.2%) | 1.24 (0.89-1.73, p=.208) |  |
| **Family genetic history** | Neither parent is myopic | 271 (80.7%) | 500 (64.8%) |  |  |
|  | Father is myopic | 26 (7.7%) | 112 (14.5%) | **2.33 (1.49-3.67, p<.001)** | **2.32 (1.46-3.69, p<.001)** |
|  | Mother is myopic | 31 (9.2%) | 115 (14.9%) | **2.01 (1.32-3.07, p=.001)** | **1.95 (1.26-3.03, p=.003)** |
|  | Both parents are myopic | 8 (2.4%) | 45 (5.8%) | **3.05 (1.42-6.56, p=.004)** | **2.87 (1.30-6.36, p=.009)** |
| **Frequency of changing seat** | Weekly | 76 (22.6%) | 127 (16.5%) |  |  |
|  | Biweekly | 63 (18.8%) | 176 (22.8%) | 1.67 (1.12-2.51, p=.013) | 1.29 (0.84-2.00, p=.244) |
|  | Once a month/no change | 197 (58.6%) | 469 (60.8%) | 1.42 (1.02-1.98, p=.035) | 1.19 (0.83-1.70, p=.343) |
| **Adjust the height of the desk and chair according to the height** | Never or non-adjustable | 188 (56%) | 468 (60.6%) |  |  |
|  | Once a year | 40 (11.9%) | 88 (11.4%) | 0.88 (0.59-1.33, p=.555) | 0.98 (0.63-1.53, p=.944) |
|  | Once a term | 59 (17.6%) | 137 (17.7%) | 0.93 (0.66-1.32, p=.696) | 1.17 (0.80-1.71, p=.411) |
|  | Once every 2 to 3 months | 49 (14.6%) | 79 (10.2%) | 0.65 (0.44-0.96, p=.031) | 0.92 (0.59-1.42, p=.692) |
| **Frequency of daily eye exercises at school** | Once | 106 (31.5%) | 191 (24.7%) |  |  |
|  | Twice and more | 164 (48.8%) | 306 (39.6%) | 1.04 (0.76-1.40, p=.822) | 1.01 (0.73-1.40, p=.953) |
|  | No | 66 (19.6%) | 275 (35.6%) | **2.31 (1.62-3.31, p<.001)** | **1.63 (1.09-2.45, p=.018)** |
| **Recess activity space** | Inside the teaching building | 183 (54.5%) | 511 (66.2%) |  |  |
|  | Outdoor | 153 (45.5%) | 261 (33.8%) | 0.61 (0.47-0.79, p<.001) | 0.77 (0.57-1.02, p=.072) |
| **Average daily time spent doing homework or reading and writing after school** | <1 h | 116 (34.5%) | 250 (32.4%) |  |  |
|  | 1-2 h | 136 (40.5%) | 300 (38.9%) | 1.02 (0.76-1.38, p=.879) |  |
|  | ≥2 h | 58 (17.3%) | 166 (21.5%) | 1.33 (0.92-1.93, p=.134) |  |
|  | No | 26 (7.7%) | 56 (7.3%) | 1.00 (0.60-1.67, p=.998) |  |
| **Average duration of extracurricular tuition per week** | No | 192 (57.1%) | 439 (56.9%) |  |  |
|  | <3 h | 120 (35.7%) | 270 (35%) | 0.98 (0.75-1.29, p=.908) |  |
|  | ≥3 h | 24 (7.1%) | 63 (8.2%) | 1.15 (0.70-1.89, p=.588) |  |
| **Read and write with your chest more than one punch away from the table** | No | 192 (57.1%) | 458 (59.3%) |  |  |
|  | Yes | 144 (42.9%) | 314 (40.7%) | 0.91 (0.71-1.18, p=.497) |  |
| **Read and write with eyes more than one foot away from the book** | No | 192 (57.1%) | 449 (58.2%) |  |  |
|  | Yes | 144 (42.9%) | 323 (41.8%) | 0.96 (0.74-1.24, p=.752) |  |
| **Read and write with fingers one inch from the tip of the pen** | No | 169 (50.3%) | 397 (51.4%) |  |  |
|  | Yes | 167 (49.7%) | 375 (48.6%) | 0.96 (0.74-1.24, p=.730) |  |
| **Average daily computer hours** | No | 156 (46.4%) | 330 (42.7%) |  |  |
|  | <1 h | 80 (23.8%) | 178 (23.1%) | 1.05 (0.76-1.46, p=.761) |  |
|  | ≥1h | 100 (29.8%) | 264 (34.2%) | 1.25 (0.93-1.68, p=.146) |  |
| **Hours of mobile electronic devices use** | No | 127 (37.8%) | 280 (36.3%) |  |  |
|  | <0.5 h | 11 (3.3%) | 17 (2.2%) | 0.70 (0.32-1.54, p=.376) |  |
|  | ≥0.5 h | 198 (58.9%) | 475 (61.5%) | 1.09 (0.83-1.42, p=.536) |  |
| **Reading a book or electronic screen in direct sunlight** | No / Occasionally | 310 (92.3%) | 695 (90%) |  |  |
|  | Often | 19 (5.7%) | 58 (7.5%) | 1.36 (0.80-2.33, p=.258) |  |
|  | Always | 7 (2.1%) | 19 (2.5%) | 1.21 (0.50-2.91, p=.669) |  |
| **Reading the electronic screen with the lights off after dark** | No / Occasionally | 290 (86.3%) | 568 (73.6%) |  |  |
|  | Often | 35 (10.4%) | 138 (17.9%) | **2.01 (1.35-2.99, p<.001)** | **2.14 (1.33-3.43, p=.002)** |
|  | Always | 11 (3.3%) | 66 (8.5%) | **3.06 (1.59-5.89, p<.001)** | **2.68 (1.13-6.39, p=.026)** |
| **Reading a book or electronic screen while lying down or lying on the back** | No / Occasionally | 252 (75%) | 506 (65.5%) |  |  |
|  | Often | 64 (19%) | 195 (25.3%) | 1.52 (1.10-2.09, p=.011) | 1.37 (0.92-2.06, p=.124) |
|  | Always | 20 (6%) | 71 (9.2%) | 1.77 (1.05-2.97, p=.031) | 0.64 (0.30-1.38, p=.258) |
| **Reading a book or electronic screen while walking or riding in a car** | No / Occasionally | 273 (81.2%) | 614 (79.5%) |  |  |
|  | Often | 54 (16.1%) | 104 (13.5%) | 0.86 (0.60-1.23, p=.396) | 1.01 (0.81-1.26, p=.918) |
|  | Always | 9 (2.7%) | 54 (7%) | 2.67 (1.30-5.48, p=.008) | 1.65 (0.63-4.29, p=.308) |
| **When using the computer, eyes from the screen more than 66cm** | No / Occasionally | 160 (47.6%) | 367 (47.5%) |  |  |
|  | Often / Always | 176 (52.4%) | 405 (52.5%) | 1.00 (0.78-1.30, p=.980) |  |
| **When using eyes at close range, how often to rest your eyes** | < 15 min | 85 (25.3%) | 169 (21.9%) |  |  |
|  | ≤ 15 < 30 min | 67 (19.9%) | 161 (20.9%) | 1.21 (0.82-1.78, p=.336) |  |
|  | ≤ 30 <60 min | 52 (15.5%) | 126 (16.3%) | 1.22 (0.80-1.85, p=.350) |  |
|  | ≥ 60 min | 132 (39.3%) | 316 (40.9%) | 1.20 (0.87-1.68, p=.271) |  |
| **Daytime outdoor activity hours** | < 1 h | 88 (26.2%) | 219 (28.4%) |  |  |
|  | 1-2 h | 136 (40.5%) | 315 (40.8%) | 0.93 (0.68-1.28, p=.659) |  |
|  | ≥ 2 h | 112 (33.3%) | 238 (30.8%) | 0.85 (0.61-1.19, p=.354) |  |
| **Average daily sleep duration** | < 8 h | 131 (39%) | 420 (54.4%) |  |  |
|  | ≥ 8 h | 205 (61%) | 352 (45.6%) | **0.54 (0.41-0.70, p<.001)** | **0.71 (0.54-0.95, p=.020)** |
| * Vocational high schools are only located in the urban area, so there is no region variable.. | | | | | |

**Supplementary Table 8 Analysis of** **associated factors for myopia among primary and secondary school students in Shenyang in 2021** **(sensitivity analysis)**

| **Variables** | **Levels** | **Non-myopia**  (N=5446) | **Myopia**  (N=14436) | **OR (****univariate)** | **OR (****multivariate)** |
| --- | --- | --- | --- | --- | --- |
| **Sex** | Male | 3093 (56.8%) | 6772 (46.9%) |  |  |
|  | Female | 2353 (43.2%) | 7664 (53.1%) | **1.49 (1.40-1.58, p<.001)** | **1.50 (1.40-1.61, p<.001)** |
| **Educational stage** | Primary 4-6th grade | 2946 (54.1%) | 4322 (29.9%) |  |  |
|  | Middle school | 1578 (29%) | 5240 (36.3%) | **2.26 (2.10-2.44, p<.001)** | **2.34 (2.15-2.55, p<.001)** |
|  | General high school | 586 (10.8%) | 4102 (28.4%) | **4.77 (4.32-5.26, p<.001)** | **4.60 (4.08-5.18, p<.001)** |
|  | Vocational high school | 336 (6.2%) | 772 (5.3%) | **1.57 (1.37-1.79, p<.001)** | **1.44 (1.22-1.69, p<.001)** |
| **Region** | Urban | 4079 (74.9%) | 10673 (73.9%) |  |  |
|  | Rural | 1367 (25.1%) | 3763 (26.1%) | 1.05 (0.98-1.13, p=.165) |  |
| **Ethnicity** | Han | 4209 (77.3%) | 11161 (77.3%) |  |  |
|  | Others | 1237 (22.7%) | 3275 (22.7%) | 1.00 (0.93-1.08, p=.967) |  |
| **Family genetic history** | Neither parent is myopic | 3769 (69.2%) | 7740 (53.6%) |  |  |
|  | Father is myopic | 514 (9.4%) | 2092 (14.5%) | **1.98 (1.79-2.20, p<.001)** | **2.17 (1.94-2.42, p<.001)** |
|  | Mother is myopic | 830 (15.2%) | 2979 (20.6%) | **1.75 (1.60-1.91, p<.001)** | **2.01 (1.83-2.20, p<.001)** |
|  | Both parents are myopic | 333 (6.1%) | 1625 (11.3%) | **2.38 (2.10-2.69, p<.001)** | **2.95 (2.59-3.37, p<.001)** |
| **Frequency of changing seat** | Weekly | 3158 (58%) | 7879 (54.6%) |  |  |
|  | Biweekly | 785 (14.4%) | 2136 (14.8%) | 1.09 (1.00-1.20, p=.064) | 1.00 (0.91-1.10, p=.995) |
|  | Once a month/no change | 1503 (27.6%) | 4421 (30.6%) | 1.18 (1.10-1.27, p<.001) | 0.95 (0.88-1.03, p=.245) |
| **Adjust the height of the desk and chair according to the height** | Never or non-adjustable | 1856 (34.1%) | 5804 (40.2%) |  |  |
|  | Once a year | 736 (13.5%) | 1794 (12.4%) | 0.78 (0.70-0.86, p<.001) | 0.92 (0.83-1.02, p=.130) |
|  | Once a term | 1569 (28.8%) | 3701 (25.6%) | 0.75 (0.70-0.82, p<.001) | 1.00 (0.91-1.09, p=.949) |
|  | Once every 2 to 3 months | 1285 (23.6%) | 3137 (21.7%) | 0.78 (0.72-0.85, p<.001) | 0.98 (0.89-1.08, p=.662) |
| **Frequency of daily eye exercises at school** | Once | 3518 (64.6%) | 9615 (66.6%) |  |  |
|  | Twice and more | 1770 (32.5%) | 4106 (28.4%) | **0.85 (0.79-0.91, p<.001)** | **0.78 (0.73-0.84, p<.001)** |
|  | No | 158 (2.9%) | 715 (5%) | 1.66 (1.39-1.98, p<.001) | 1.13 (0.92-1.38, p=.244) |
| **Recess activity space** | Inside the teaching building | 1236 (22.7%) | 4474 (31%) |  |  |
|  | Outdoor | 4210 (77.3%) | 9962 (69%) | **0.65 (0.61-0.70, p<.001)** | **0.85 (0.78-0.93, p<.001)** |
| **Average daily time spent doing homework or reading and writing after school** | No | 461 (8.5%) | 705 (4.9%) |  |  |
|  | <1 h | 2066 (37.9%) | 4436 (30.7%) | **1.40 (1.23-1.60, p<.001)** | **1.22 (1.06-1.40, p=.005)** |
|  | 1-2 h | 2016 (37%) | 5663 (39.2%) | **1.84 (1.62-2.09, p<.001)** | **1.23 (1.07-1.42, p=.003)** |
|  | ≥2 h | 903 (16.6%) | 3632 (25.2%) | **2.56 (2.21-2.96, p<.001)** | **1.41 (1.20-1.65, p<.001)** |
| **Average duration of extracurricular tuition per week** | No | 2424 (44.5%) | 6457 (44.7%) |  |  |
|  | <3 h | 2317 (42.5%) | 5883 (40.8%) | 0.95 (0.89-1.02, p=.161) | 0.97 (0.90-1.05, p=.473) |
|  | ≥3 h | 705 (12.9%) | 2096 (14.5%) | 1.12 (1.01-1.23, p=.027) | 0.91 (0.81-1.01, p=.074) |
| **Read and write with your chest more than one punch away from the table** | No | 2385 (43.8%) | 6935 (48%) |  |  |
|  | Yes | 3061 (56.2%) | 7501 (52%) | 0.84 (0.79-0.90, p<.001) | 1.10 (1.00-1.20, p=.052) |
| **Read and write with eyes more than one foot away from the book** | No | 2158 (39.6%) | 6781 (47%) |  |  |
|  | Yes | 3288 (60.4%) | 7655 (53%) | **0.74 (0.70-0.79, p<.001)** | **0.75 (0.68-0.83, p<.001)** |
| **Read and write with fingers one inch from the tip of the pen** | No | 1878 (34.5%) | 5425 (37.6%) |  |  |
|  | Yes | 3568 (65.5%) | 9011 (62.4%) | 0.87 (0.82-0.93, p<.001) | 1.09 (0.99-1.19, p=.066) |
| **Average daily computer hours** | No | 3104 (57%) | 8205 (56.8%) |  |  |
|  | <1 h | 1583 (29.1%) | 4067 (28.2%) | 0.97 (0.91-1.04, p=.434) |  |
|  | ≥1h | 759 (13.9%) | 2164 (15%) | 1.08 (0.98-1.18, p=.109) |  |
| **mobile electronic devices use** | No | 1848 (33.9%) | 4528 (31.4%) |  |  |
|  | Yes | 3598 (66.1%) | 9908 (68.6%) | 1.12 (1.05-1.20, p<.001) | 1.00 (0.93-1.08, p=.983) |
| **Reading a book or electronic screen in direct sunlight** | No / Occasionally | 5192 (95.3%) | 13674 (94.7%) |  |  |
|  | Often | 192 (3.5%) | 562 (3.9%) | 1.11 (0.94-1.31, p=.215) |  |
|  | Always | 62 (1.1%) | 200 (1.4%) | 1.22 (0.92-1.63, p=.166) |  |
| **Reading the electronic screen with the lights off after dark** | No / Occasionally | 5077 (93.2%) | 13095 (90.7%) |  |  |
|  | Often | 247 (4.5%) | 925 (6.4%) | 1.45 (1.26-1.68, p<.001) | 1.07 (0.91-1.27, p=.415) |
|  | Always | 122 (2.2%) | 416 (2.9%) | 1.32 (1.08-1.62, p=.007) | 1.11 (0.88-1.42, p=.379) |
| **Reading a book or electronic screen while lying down or lying on the back** | No / Occasionally | 4694 (86.2%) | 11866 (82.2%) |  |  |
|  | Often | 626 (11.5%) | 2157 (14.9%) | 1.36 (1.24-1.50, p<.001) | 1.08 (0.97-1.21, p=.158) |
|  | Always | 126 (2.3%) | 413 (2.9%) | 1.30 (1.06-1.59, p=.012) | 0.89 (0.69-1.14, p=.357) |
| **Reading a book or electronic screen while walking or riding in a car** | No / Occasionally | 5177 (95.1%) | 13372 (92.6%) |  |  |
|  | Often | 219 (4%) | 842 (5.8%) | 1.49 (1.28-1.73, p<.001) | 0.83 (0.69-1.05, p=.051) |
|  | Always | 50 (0.9%) | 222 (1.5%) | 1.72 (1.26-2.34, p<.001) | 1.26 (0.86-1.84, p=.240) |
| **When using the computer, eyes from the screen more than 66cm** | No / Occasionally | 1620 (29.7%) | 4801 (33.3%) |  |  |
|  | Often / Always | 3826 (70.3%) | 9635 (66.7%) | 0.85 (0.79-0.91, p<.001) | 0.97 (0.90-1.05, p=.467) |
| **When using eyes at close range, how often to rest your eyes** | < 15 min | 1405 (25.8%) | 3355 (23.2%) |  |  |
|  | ≤ 15 < 30 min | 1718 (31.5%) | 4649 (32.2%) | 1.13 (1.04-1.23, p=.003) | 1.03 (0.94-1.13, p=.516) |
|  | ≤ 30 <60 min | 835 (15.3%) | 2669 (18.5%) | 1.34 (1.21-1.48, p<.001) | 1.06 (0.95-1.18, p=.277) |
|  | ≥ 60 min | 1488 (27.3%) | 3763 (26.1%) | 1.06 (0.97-1.15, p=.194) | 0.94 (0.86-1.03, p=.205) |
| **Daytime outdoor activity hours** | < 1 h | 1295 (23.8%) | 4088 (28.3%) |  |  |
|  | 1-2 h | 2293 (42.1%) | 5942 (41.2%) | 0.82 (0.76-0.89, p<.001) | 0.92 (0.85-1.01, p=.075) |
|  | ≥ 2 h | 1858 (34.1%) | 4406 (30.5%) | 0.75 (0.69-0.82, p<.001) | 0.92 (0.84-1.00, p=.060) |
| **Average daily sleep duration** | < 8 h | 1596 (29.3%) | 6739 (46.7%) |  |  |
|  | ≥ 8 h | 3850 (70.7%) | 7697 (53.3%) | **0.47 (0.44-0.51, p<.001)** | **0.91 (0.84-0.99, p=.027)** |

**Supplementary Figure**


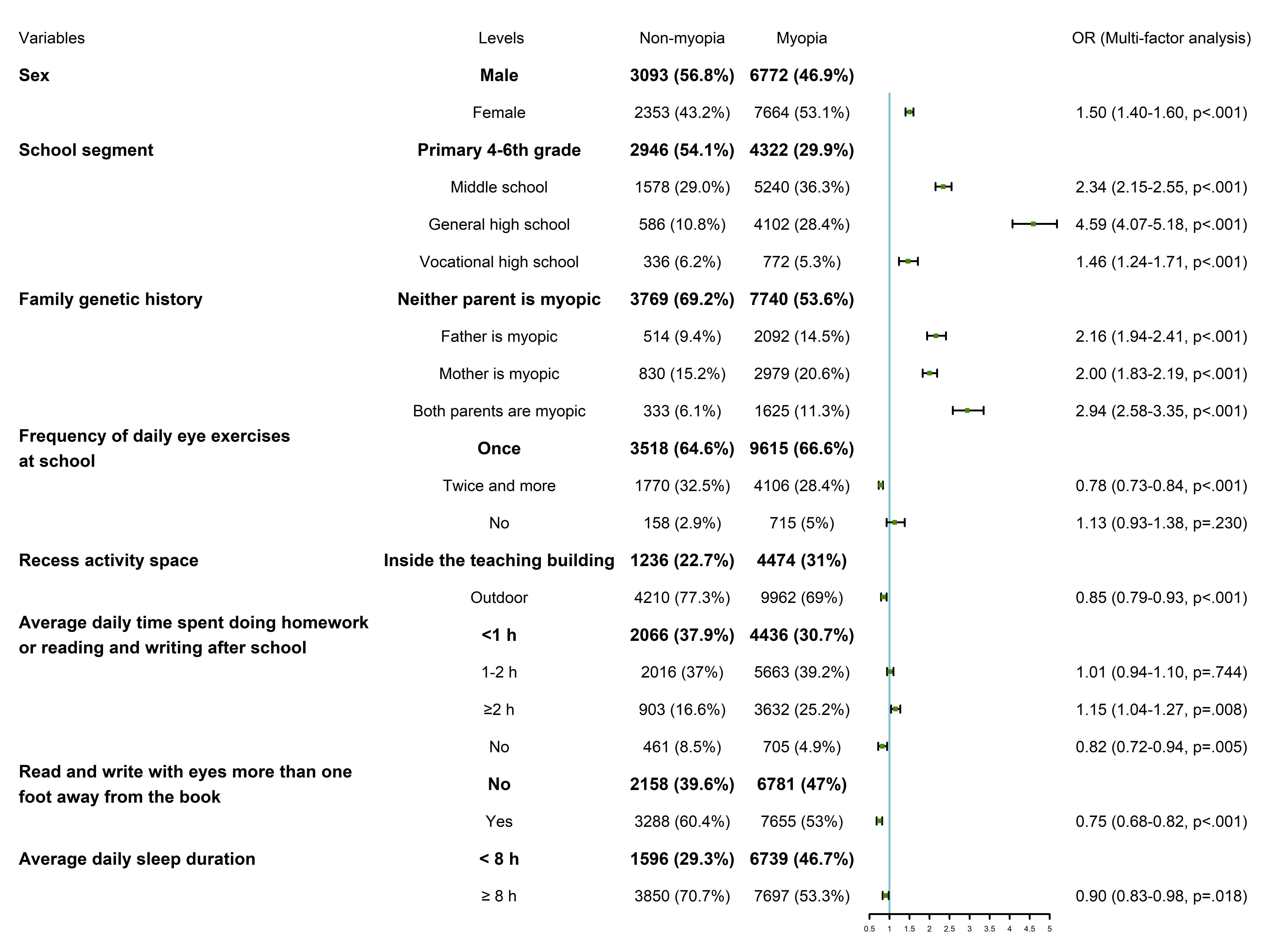


**Supplementary Figure 1** Forest plot of multivariate analysis results.
